# Summarizing clinical evidence utilizing large language models for cancer treatments: a blinded comparative analysis

**DOI:** 10.3389/fdgth.2025.1569554

**Published:** 2025-04-29

**Authors:** Samuel Rubinstein, Aleenah Mohsin, Rahul Banerjee, Will Ma, Sanjay Mishra, Mary Kwok, Peter Yang, Jeremy L. Warner, Andrew J. Cowan

**Affiliations:** ^1^ Division of Hematology, Department of Medicine, University of North Carolina, Chapel Hill, NC, United States; ^2^ Brown University Health Cancer Institute, Rhode Island Hospital, Providence, RI, United States; ^3^ Division of Hematology-Oncology, University of Washington, Seattle, WA, United States; ^4^ Clinical Research Division, Fred Hutch Cancer Center, Seattle, WA, United States; ^5^ Hope AI, Inc, Princeton, NJ, United States; ^6^ Department of Medicine, Massachusetts General Hospital, Boston, MA, United States; ^7^ Legorreta Cancer Center, Brown University, Providence, RI, United States

**Keywords:** large language models, clinical evidence summarization, cancer treatment synopses, multiple myeloma, comparative analysis

## Abstract

**Background:**

Concise synopses of clinical evidence support treatment decision-making but are time-consuming to curate. Large language models (LLMs) offer potential but they may provide inaccurate information. We objectively assessed the abilities of four commercially available LLMs to generate synopses for six treatment regimens in multiple myeloma and amyloid light chain (AL) amyloidosis.

**Methods:**

We compared the performance of four LLMs: Claude 3.5, ChatGPT 4.0; Gemini 1.0 and Llama-3.1. Each LLM was prompted to write synopses for six regimens. Two hematologists independently assessed accuracy, completeness, relevance, clarity, coherence, and hallucinations using Likert scales. Mean scores with 95% confidence intervals (CI) were calculated across all domains and inter-rater reliability was evaluated using Cohen's quadratic weighted kappa.

**Results:**

Claude demonstrated the highest performance in all domains, outperforming the other LLMs in accuracy: mean Likert score 3.92 (95% CI 3.54–4.29); ChatGPT 3.25 (2.76–3.74); Gemini 3.17 (2.54–3.80); Llama 1.92 (1.41–2.43);completeness: mean Likert score 4.00 (3.66–4.34); GPT 2.58 (2.02–3.15); Gemini 2.58 (2.02–3.15); Llama 1.67 (1.39–1.95); and extentofhallucinations: mean Likert score 4.00 (4.00–4.00); ChatGPT 2.75 (2.06-3.44); Gemini 3.25 (2.65–3.85); Llama 1.92 (1.26–2.57). Llama performed considerably poorer across all the studied domains. ChatGPT and Gemini had intermediate performance. Notably, none of the LLMs registered perfect accuracy, completeness, or relevance.

**Conclusion:**

Claude performed at a consistently higher level than other LLMs, all tested LLMs required careful editing from a domain expert to become usable. More time will be needed to determine the suitability of LLMsto independently generate clinical synopses.

## Introduction

Summarizing clinical evidence is a critical task for guideline developers and clinicians. These summaries can take many forms: lengthy technical reports (e.g., systematic reviews and guidelines), limited clinical briefs on physician-facing media and websites, and concise point-of- care synopses. The latter are increasingly desirable in busy clinical settings because they can quickly inform clinical decision-making without forcing physicians to manually distill an ever- growing base of medical literature. Given the sheer volume of literature published each year, manual curation of relevant data for synopses is time-consuming and labor-intensive (1). Regularly monitoring new developments to update the synopses and filtering through studies to avoid repetition and contradictions to produce reliable synopses takes significant time and effort, which is often not feasible in clinical settings.

Large language models (LLMs) hold considerable potential in this context. LLMs can process large amounts of data relatively quickly using artificial intelligence, thereby reducing the time needed to curate and summarize contemporary literature manually (2, 3). Recent studies have evaluated the quality, accuracy, and potential biases in summaries generated by LLMs in biomedical domains. Interestingly, some findings suggest that large language models can outperform medical experts when it comes to summarizing clinical texts (4). One study specifically examined how ChatGPT-4 performs in generating lay summaries of scientific abstracts. Among 34 volunteers, 85.3% found the AI-generated summaries were more accessible than the original abstracts, and 73.5% considered them more transparent than the original abstracts. Importantly, none of the summaries were perceived as harmful (5). However, other assessments have flagged ongoing issues. ChatGPT's summaries were generally easy to read, but concerns remain around factual accuracy and the exclusion of key details (6).

The current generation of LLMs remains prone to errors and hallucinations. Specifically, LLMs may generate coherent-sounding information that in actuality may be factually incorrect, fabricated, and/or irrelevant (7). Additionally, LLMs may not always grasp the nuances and complexities of information in clinical context, which might lead to oversimplified synopses. These shortcomings not only undermine the overall reliability of curated information but could also be harmful for patients if not accurate or properly contextualized (8). As a hypothetical example, an LLM-generated synopsis for transplant-ineligible newly diagnosed multiple myeloma (MM) may identify quadruplet induction as the standard of care based on recent trials without adding that older and frailer patients (for whom quadruplet induction may be inappropriate) were excluded from these trials (9).

Given these uncertainties, it is unclear whether the benefits of using LLMs for formulation of synopses in oncology outweigh the risks. The performance of LLMs to generate concise synopses of the evidence supporting cancer treatment has not been previously analyzed. Our evaluation is the first of its kind to assess the capabilities of widely available LLMs. We aimed to objectively assess and compare the abilities of four commercial LLMs to generate reliable and clinically useful synopses for six treatment regimens in MM and AL amyloidosis.

## Methods

HemOnc.org is an online, freely accessible collaborative wiki of cancer drug and blood disorder treatment information. Developed since 11/2011, it provides fully referenced drug and regimen information, including granular dosing and administration details (10). Curated by domain experts, details presented on HemOnc.org are highly technical and concise, with the aim of helping healthcare professionals find the information they need, quickly. As HemOnc.org has grown in scope and audience, lay summaries for learners, patients, and caregivers have become increasingly necessary. To address the need to better contextualize individual cancer therapies, we began manually developing synopses of different treatment regimens in 2021. Page editors, usually experts in their specific disease, oversee development of these, which takes considerable time and effort.

In 2023, with the widespread advancement and proliferation of LLMs, we developed an LLM pilot program, utilizing LLMs to generate human-readable synopses of some of the most relevant anti- cancer treatment regimens on HemOnc.org, overseen by the page editors. To better understand the performance of LLMs, we prospectively evaluated LLM-generated synopses for several widely used MM and AL amyloidosis treatment regimens. We selected these two similar yet clinically distinct diseases as they are among the most widely searched diseases on HemOnc.org.

We tested the performance of four commercially available LLMs: Claude 3.5 (“Claude”), ChatGPT 4.0 (“ChatGPT”), Gemini 1.0 (“Gemini”), and Llama 2 (“Llama”). Synopses were created for the following MM and AL amyloidosis treatment regimens:

1. Daratumumab, lenalidomide, bortezomib, and dexamethasone (Dara-VRd) (11, 12)
2. Carfilzomib, lenalidomide, and dexamethasone (KRd) (13)
3. Bortezomib, thalidomide, dexamethasone, cisplatin, doxorubicin (Adriamycin), cyclophosphamide, and etoposide (VTD-PACE) (14)
4. Daratumumab, cyclophosphamide, bortezomib, and dexamethasone (Dara-CyBorD) (15)
5. Elranatamab monotherapy (16)
6. Talquetamab monotherapy (17)

We formulated the prompts in plain language, similar to how a clinician would ask a question, reflecting a *zero shot prompting* strategy: “Write a synopsis for the development and evolution of therapy with [Drug Regimen] for [Diagnosis—Multiple Myeloma or Amyloidosis]. Use citations from the literature.” We used a single prompt for each question, without deploying multiple rephrasings or other variants. Models were accessed using the user interface by AJC. No prompt tuning or iterative engineering was performed and all the responses reflect the default model behavior. A full listing of the prompts and outputs from each LLM is provided in the Supplementary Materials.

The generated synopses were then assessed by two board-certified hematologists specializing in the treatment of MM and AL amyloidosis (RB/SR). The evaluation process was completed using a REDCap (Research Electronic Data Capture) survey at University of Washington (Institute of Translational Health Sciences) (18). Reviewers evaluated the synopses using a 5-point Likert scale across five criteria: accuracy, completeness, relevance, clarity, and coherence, while hallucinations were assessed on a 4-point ordinal scale. A traditional 5-point Likert scale was not used, as a “neutral” midpoint held limited interpretive value in this context. The scale was defined as follows:

1 = Many hallucinations,
2 = Some hallucinations,
3 = Few hallucinations,
4 = No hallucinations.

A separate question asked whether the synopsis would require only minimal editing, and a section for narrative comments on the LLM output was included. (Supplementary Materials).

Data analysis was performed using R version 4.4.1 (2024-06-14). Mean scores for each LLM across all regimens and criteria to assess overall performance were calculated. Lower scores corresponded to lower performance, while higher scores corresponded to higher performance. Inter-rater reliability was assessed using Cohen's quadratic weighted kappa to evaluate agreement between reviewers across criteria and regimens. Additionally, the proportion of synopses requiring minimal editing was analyzed. Visualizations, including bar plots and heatmaps, were created using ggplot2 to illustrate the comparative performance of LLMs across different criteria and regimens.

## Results

### Overall performance

**Figure 1:**
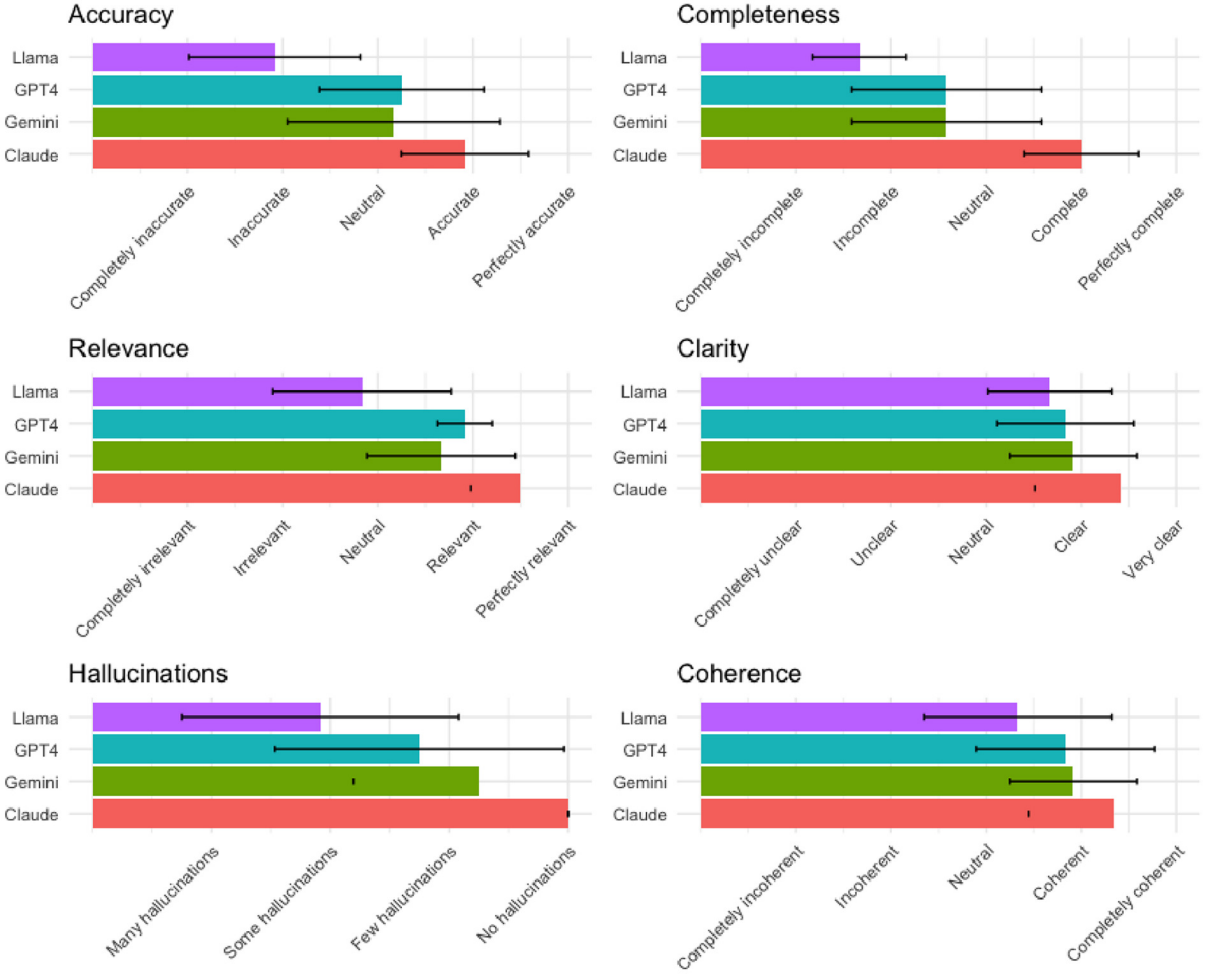
Summary of performance of each LLM by criterion.

A summary of LLM performance by criterion is shown in Table 1; Figure 1. Overall, none of the tested LLMs performed consistently across domains. Of the LLMs, Claude performed consistently better than GPT4, Gemini, and Llama in all domains (Mean Scores: accuracy 3.9 [95% CI 3.54–4.29], completeness 4.0 [95% CI 3.66–4.34], relevance 4.5 [95% CI 4.2–4.8], clarity 4.4 [95% CI 3.91–4.93], hallucinations 4.0 [95% CI 4–4], and coherence 3.8 [95% CI 3.83–4.84]). Llama consistently had the lowest mean Likert scores, and GPT4 and Gemini largely performed similarly between Claude and GPT4.

**Table 1:** A summary of LLM performance by criterion (mean with 95% CI).

Criterion	Claude	GPT4	Gemini	Llama
Accuracy	3.92 (3.54–4.29)	3.25 (2.76–3.74)	3.17 (2.54–3.80)	1.92 (1.41–2.43)
Completeness	4.00 (3.66–4.34)	2.58 (2.02–3.15)	2.58 (2.02–3.15)	1.67 (1.39–1.95)
Relevance	4.50 (4.20–4.80)	3.92 (3.75–4.08)	3.67 (3.23–4.11)	2.83 (2.30–3.36)
Clarity	4.42 (3.91–4.93)	3.83 (3.43–4.24)	3.92 (3.54–4.29)	3.67 (3.30–4.04)
Hallucinations	4.00 (4.00–4.00)	2.75 (2.06–3.44)	3.25 (2.65–3.85)	1.92 (1.26–2.58)
Coherence	4.33 (3.83–4.84)	3.83 (3.30–4.36)	3.92 (3.54–4.29)	3.33 (2.78–3.89)

Only Claude performed routinely well in the domain of hallucinations, with minimal to no hallucinations detected by the reviewers. Regarding the need for corrective edits (Table 2), Claude appeared to perform the best overall, with 66.7% of synopses requiring minimal editing, while Gemini and Llama performed poorly, with only 16.7% and 8.3% requiring minimal editing, respectively.

**Table 2:** Percentage of synopses requiring minimal editing (with 95% CI).

LLM	Result
Claude	66.7% (38.8%–94.5%)
GPT4	33.3% (5.5%–61.2%)
Gemini	16.7% (0.0%–38.7%)
Llama	8.3% (0.0%–24.7%)

### Inter-rater reliability

Inter-rater reliability varied considerably across criteria and regimens (Table 3). Overall agreement was moderate for accuracy (*κ* = 0.649) and relevance (*κ* = 0.761), fair for completeness (*κ* = 0.521), and poor-to-fair for hallucinations (*κ* = 0.362), coherence (*κ* = 0.353), and clarity (*κ* = 0.135). Agreement was generally strongest for the Dara-VRd and Dara-CyBorD regimens, with perfect agreement on relevance (*κ* = 1.0) and substantial agreement on accuracy (*κ* = 0.75) for both. In contrast, agreement was weaker for newer agents like talquetamab, where negative/zero kappa values were observed for accuracy and relevance. The KRd regimen showed strong agreement across most domains, particularly for completeness (*κ* = 0.81) and accuracy (*κ* = 0.8).

**Table 3:** Cohen's weighted kappa by regimen and criterion.

Criterion	All regimens	Dara-VRd	Dara-CyBo rD	Elranata mab	KRd	Talquet a mab	VTD-PACE
Accuracy	0.649	0.75	0.75	0.7	0.8	−0.125	0.667
Completeness	0.521	0.667	0.667	0.111	0.81	0.417	0.444
Relevance	0.761	1	1	1	0.8	0	0.667
Clarity	0.135	0.2	0.556	−0.3	0.5	0	0.25
Hallucination	0.362	0.643					
0.75	0.312	0	0.769	0.667	0		
Coherence	0.353		0.667	−0.667	0	NA	0.5

Dara-VRd, daratumumab, lenalidomide, bortezomib, and dexamethasone; Dara-CyBorD, daratumumab, cyclophosphamide, bortezomib, and dexamethasone; KRd, carfilzomib, lenalidomide, and dexamethasone; VTD-PACE, bortezomib, thalidomide, dexamethasone, cisplatin, doxorubicin, cyclophosphamide, and etoposide.

### Qualitative insights

Overall, several themes emerged from the narrative comments provided by reviewers (Supplementary Material). Many comments highlighted inaccuracies in LLM-generated synopses, particularly clinical trial names, purpose, and results. The reviewers also noted missing information and lack of detail on key aspects of clinical trials. Safety information was also highlighted as a deficiency in many comments across regimens and LLMs. Citations were noted to be frequently incorrect, or references were missing entirely. Occasionally, non-existent (i.e., hallucinated) studies were cited by LLMs.

Reviewers also indicated the presence of language patterns that are characteristic of AI-generated text, such as flowery language or generic statements. Specific factual inaccuracies further underscored the limitations of the models. For instance, GPT-4 incorrectly cited the GEM-CESAR trial, which is neither an NDMM (newly diagnosed multiple myeloma) study nor one evaluating the Dara-RVd regimen. Similarly, CASTOR, a trial conducted in the relapsed/refractory setting was included by Chatgpt despite its irrelevance to frontline therapy. Conversely, PERSEUS, a key trial directly investigating Dara-RVd in NDMM was omitted. Moreover, LLaMA inaccurately stated that KRd demonstrated “similar efficacy but improved tolerability” compared to VRd in the ENDURANCE trial. This interpretation is misleading, as the trial did not show superiority of KRd in efficacy or tolerability. These errors suggest a lack of specificity in identifying appropriate evidence and reinforce the importance of expert review.

## Discussion

The rapid development and accessibility of LLMs has the promise to revolutionize knowledge curation across domains, including medicine. The challenge of digesting and concatenating a high volume of primary medical literature into interpretations which are both usable by, and useful to, increasingly busy clinicians is immense.

Before the LLM era, this process typically took place in guideline committees led by experts or through review articles commissioned by high-impact journals. Deploying LLMs to supplement these processes has the potential to repurpose experts’ time towards primary investigation and avoid conflicts of interest (19, 20). At present, it is not clear whether these potential advantages of using LLMs in medical knowledge curation outweigh the disadvantages. Currently available LLMs remain deficient at accurately identifying citations to support their assertions and remain prone to hallucinations (21). As medical knowledge curation is fundamentally used to support clinical decisions, these shortcomings could be catastrophic to patients. Before being widely deployed clinically, LLMs need to be rigorously evaluated to minimize the potential for harm.

To our knowledge, this is the first evaluation of widely available LLMs for the task of evidence summarization in oncology. The limited literature pertaining to scientific literature summarization suggests a potential beneficial role. In a recent publication, investigators assessed the ability of ChatGPT to summarize 140 peer reviewed abstracts from 14 journals; the generated summaries were found to be shorter than the abstracts and were felt by reviewers to be of sufficient quality, accuracy, and without bias (6). In a separate study, ChatGPT4 was used to generate lay summaries of scientific abstracts which were assessed by reviewers (5). The analysis found that the summaries rated high for accuracy and relevance, and none were deemed to be harmful. Another recent analysis showed that ChatGPT-4 demonstrates superior performance over LLaMA across three key NLP tasks; text summarization, data analysis, and question answering and achieved higher accuracy, coherence, and relevance (22). In our study, we find wide variation across LLMs in accuracy, completeness, relevance, clarity and coherence and hallucinations. Interestingly, the performance of each LLM was relatively consistent across all examined domains: Llama-2 performed worst, GPT4 and Gemini had middling performance, while Claude consistently outperformed the other LLMs. Most encouragingly, Claude was not observed to hallucinate by either expert reviewer across all six synopses. A recent study also highlighted that Claude generated the most human-like summaries, but Gemini models stood out for their efficiency and cost-effectiveness (23). Avoiding the dissemination of entirely fabricated citations is a critical bar for LLMs to clear prior to widespread deployment in medical knowledge curation.

Although Claude had the most encouraging performance, it still fell short of meeting the necessary quality standard for generating synopses usable in clinical medicine, performing worst in the domain of accuracy. Arguably, the increased coherence and relevance of Claude could present inaccurate information in a maximally believable way to clinicians. Nearly perfect cross- domain performance should be considered the standard for LLMs intended for application to medical literature. Furthermore, domain expert comments reveal that the synopses generated by Claude often minimized or omitted evidence concerning the toxicity associated with a given chemotherapy regimen. For other models, common error themes included incorrect or hallucinated citations, omission of critical safety data, and superficial descriptions of clinical trials. Understanding these error types can inform more targeted prompt engineering and model selection. Given the importance of safety in making treatment decisions, this minimizes the utility of these synopses to clinicians treating cancer patients. At this point, none of the other three LLMs evaluated could be recommended in place of Claude; however, it is likely that ensemble approaches or agentic approaches may overcome the limitations of a single LLM. Previously, a study has introduced the SliSum strategy which enhances summarization faithfulness in LLMs. It reduced hallucinations in models like LLaMA-2, Claude-2, and GPT-3.5 for both short and long texts without requiring additional resources (24). Fine-tuning and implementation of retrieval-augmented generation (RAG) architecture may also address some of the shortcomings yet require specific expertise and are expensive to implement.

Inter-rater reliability agreement between reviewers varied considerably. The agreement was stronger for well-established regimens like Dara-VRd and Dara-CyBorD, particularly for accuracy and relevance, where negative or zero kappa values were observed. This lack of consensus likely reflects the evolving nature of evidence for newer therapies and availability of standardized evidence for established regimens. LLMs may, thus, currently be more useful for summarizing evidence related to widely accepted therapies. It is also important to note that domains like clarity and hallucinations exhibited consistently low inter-rater reliability, irrespective of type of regimen, alluding to the subjective nature of these criteria, nuance in understanding, and familiarity of the reviewers with the existing literature.

LLMs currently face several limitations that limit their clinical utility. Cost-efficiency and scalability remain major issues for many institutions given the high computational demands and maintenance requirements. Most LLMs are trained on static or outdated data, so they often miss the latest clinical trial findings, a serious limitation in fast-moving fields such as oncology. Accuracy, trust, and interpretability issues are compounded by the limited context awareness of LLMs, limiting their applicability to nuanced clinical scenarios and potentially leading to misleading or even unsafe recommendations. Moreover, LLMs can generate plausible-sounding but factually incorrect information (hallucinations), including inaccurate drug regimens, trial results, or citations. This poses a significant patient safety risk. Given the high stakes of medical decision-making, current deployment of LLMs must be cautious and regulated. LLMs should be restricted to augmentative roles within hybrid workflows.

Our analysis has some limitations. Although many synopses generated for this study were not usable without any editing, they may still save experts significant time in curating literature. We did not compare the time spent by experts to summarize the supporting literature of clinical regimens with and without LLMs. Furthermore, it was not feasible to assess the performance of different iterations of the same LLM (for example, GPT3 and GPT4).

Given the pace of advancements in LLM technology, advances in their capabilities to summarize medical literature may improve rapidly over time. Beyond summarization tasks, the integration of LLMs into healthcare IT infrastructure, particularly electronic health record (EHR) systems, presents a significant opportunity to streamline clinical workflows. Future research should explore the development of specialized, domain-adapted LLMs trained on curated clinical corpora and real-world patient data, which would enhance performance in nuanced tasks such as therapeutic decision-making.

## Conclusion

Despite encouraging individual aspects of LLM performances, the tested LLMs remain incapable of generating usable synopses supporting treatment regimens widely used to treat plasma cell disorders without significant input from domain experts. Their inability to incorporate real-time updates restricts the inclusion of recently published trials and therefore issues such as inaccurate citations and hallucination remain prevalent, which is especially true for fields like oncology. Moreover, they lack the nuanced clinical judgment which is required to account for patient-specific variables. Fine-tuning and implementation of retrieval-augmented generation (RAG) may also address some of the shortcomings but it requires specific expertise.

## Data Availability

The original contributions presented in the study are included in the article/Supplementary Material, further inquiries can be directed to the corresponding author.
