# Supplementary material for: Summarizing clinical evidence utilizing large language models for cancer treatments: a blinded comparative analysis
Source: Front Digit Health. 2025 Apr 29;7:1569554. doi: 10.3389/fdgth.2025.1569554 (PMC12069342; doi:10.3389/fdgth.2025.1569554)
Supplement: Supplementary file 1 [file Table1.docx]

***Supplemental Materials***

[**Large Language Model Outputs for Treatment Synopses** 2](#_heading=h.gjdgxs)

[Daratumumab, Lenalidomide, Bortezomib, and Dexamethasone (D-RVd) 2](#_heading=h.30j0zll)

[Carfilzomib, Lenalidomide, and Dexamethasone (KRd) 6](#_heading=h.1fob9te)

[VTD-PACE 11](#_heading=h.3znysh7)

[Dara-VCD 17](#_heading=h.2et92p0)

[Elranatamab 22](#_heading=h.tyjcwt)

[Talquetamab 27](#_heading=h.3dy6vkm)

Survey Tool for LLM Output Evaluation [3](#_heading=h.1t3h5sf)2

[Narrative Comments 33](#_heading=h.1t3h5sf)

# **Large Language Model Outputs for Treatment Synopses**

## Daratumumab, Lenalidomide, Bortezomib, and Dexamethasone (D-RVd)

1. **Claude 3.5 Output**

Here's a synopsis of the development and evolution of therapy using daratumumab, lenalidomide, bortezomib, and dexamethasone for newly diagnosed multiple myeloma:

The treatment landscape for newly diagnosed multiple myeloma (NDMM) has evolved significantly over the past decade, with the introduction of novel agents leading to improved outcomes. The combination of daratumumab, lenalidomide, bortezomib, and dexamethasone (D-RVd) has emerged as a highly effective regimen for NDMM patients.

Initially, the triplet regimen of lenalidomide, bortezomib, and dexamethasone (RVd) became a standard of care for NDMM based on the SWOG S0777 trial, which demonstrated superior progression-free survival (PFS) and overall survival (OS) compared to lenalidomide and dexamethasone alone [1].

Daratumumab, a monoclonal antibody targeting CD38, was first approved for relapsed/refractory multiple myeloma. Its efficacy in this setting led to investigations in NDMM. The MAIA trial showed that adding daratumumab to lenalidomide and dexamethasone significantly improved PFS in transplant-ineligible NDMM patients [2].

The GRIFFIN study was a pivotal trial evaluating the addition of daratumumab to RVd in transplant-eligible NDMM patients. This phase 2 randomized study demonstrated superior stringent complete response rates and minimal residual disease (MRD) negativity with D-RVd compared to RVd alone [3].

Long-term follow-up of the GRIFFIN trial has continued to show sustained benefits of D-RVd, with improved PFS and deeper responses [4]. These results have established D-RVd as a highly effective induction regimen for transplant-eligible NDMM patients.

For transplant-ineligible patients, the PERSEUS trial investigated D-RVd versus RVd. Preliminary results presented at ASH 2023 showed significant improvements in PFS and MRD negativity rates with D-RVd [5].

The evolution of this regimen has also included efforts to optimize its administration. Studies have explored subcutaneous formulations of daratumumab and bortezomib to improve patient convenience and reduce infusion-related reactions [6].

Ongoing research is focusing on identifying biomarkers for patient selection, optimizing treatment duration, and investigating the role of D-RVd in maintenance therapy.

In conclusion, the development of the D-RVd regimen represents a significant advance in the treatment of NDMM, offering improved depth and duration of response across patient subgroups. Its evolution continues as researchers refine its use and explore combination strategies with emerging therapies.

Would you like me to elaborate on any specific aspect of this synopsis or provide more information about the individual components of the regimen?

1. **ChatGPT 4.0 Output**

Synopsis for the Development and Evolution of Therapy with Daratumumab, Lenalidomide, Bortezomib, and Dexamethasone for Newly Diagnosed Multiple Myeloma

Introduction

The treatment landscape for multiple myeloma has significantly evolved over the past decade, driven by the introduction of novel agents and combination therapies. Among the most notable advancements is the use of daratumumab, a monoclonal antibody targeting CD38, in combination with lenalidomide, bortezomib, and dexamethasone (DRd and DVd regimens). This synopsis outlines the development and clinical evolution of these therapies, with references to key clinical trials and studies.

**Development of Key Therapies**

1. **Daratumumab (D)**

• **Mechanism of Action**: Daratumumab is a human IgG1κ monoclonal antibody that binds to CD38, inducing tumor cell death through multiple immune-mediated mechanisms, including complement-dependent cytotoxicity (CDC), antibody-dependent cellular phagocytosis (ADCP), and antibody-dependent cellular cytotoxicity (ADCC).

• **Clinical Trials**: Initial studies demonstrated significant efficacy of daratumumab as a monotherapy in heavily pretreated multiple myeloma patients (Lokhorst et al., 2015) .

2. **Lenalidomide (R)**

• **Mechanism of Action**: Lenalidomide is an immunomodulatory drug (IMiD) that enhances T-cell and natural killer (NK) cell function, inhibits angiogenesis, and directly induces apoptosis of myeloma cells.

• **Clinical Trials**: The combination of lenalidomide and dexamethasone (Rd) has been a backbone therapy for multiple myeloma, showing improved survival rates in newly diagnosed patients (Weber et al., 2007) .

3. **Bortezomib (V)**

• **Mechanism of Action**: Bortezomib is a proteasome inhibitor that disrupts the ubiquitin-proteasome pathway, leading to the accumulation of misfolded proteins and induction of apoptosis in myeloma cells.

• **Clinical Trials**: Bortezomib combined with lenalidomide and dexamethasone (VRd) has shown significant efficacy in newly diagnosed multiple myeloma patients (Richardson et al., 2010) .

4. **Dexamethasone (d)**

• **Mechanism of Action**: Dexamethasone is a corticosteroid that induces apoptosis of lymphoid cells, including myeloma cells, and has anti-inflammatory properties.

• **Clinical Trials**: Dexamethasone is used in combination with other agents to enhance their therapeutic effects and manage side effects.

**Evolution of Combination Therapies**

1. **Daratumumab, Lenalidomide, and Dexamethasone (DRd)**

• **MAIA Trial**: The MAIA trial established the efficacy of DRd in newly diagnosed multiple myeloma patients who are ineligible for autologous stem cell transplantation (ASCT). The trial demonstrated significant improvements in progression-free survival (PFS) and overall response rates (ORR) compared to Rd alone (Facon et al., 2019) .

2. **Daratumumab, Bortezomib, and Dexamethasone (DVd)**

• **CASTOR Trial**: The CASTOR trial evaluated the combination of DVd in relapsed or refractory multiple myeloma, showing enhanced PFS and ORR compared to Vd alone (Palumbo et al., 2016) . Subsequent studies have explored its use in the frontline setting.

3. **Daratumumab, Bortezomib, Lenalidomide, and Dexamethasone (DVRd)**

• **GEM-CESAR and GRIFFIN Trials**: These trials investigated DVRd in newly diagnosed patients, particularly those eligible for ASCT. Both trials reported high rates of stringent complete response (sCR) and minimal residual disease (MRD) negativity, establishing DVRd as a potent induction regimen (Mateos et al., 2020; Voorhees et al., 2020) .

**Conclusion**

The incorporation of daratumumab into combination regimens with lenalidomide, bortezomib, and dexamethasone has transformed the treatment paradigm for newly diagnosed multiple myeloma. Clinical trials have consistently demonstrated superior efficacy and deeper responses with these combinations, paving the way for improved patient outcomes. Ongoing research continues to refine these regimens and explore their long-term benefits and safety profiles.

**References:**

1. Lokhorst, H. M., Plesner, T., Laubach, J. P., et al. (2015). Targeting CD38 with daratumumab monotherapy in multiple myeloma. *New England Journal of Medicine*, 373(13), 1207-1219.

2. Weber, D. M., Chen, C., Niesvizky, R., et al. (2007). Lenalidomide plus dexamethasone for relapsed multiple myeloma in North America. *New England Journal of Medicine*, 357(21), 2133-2142.

3. Richardson, P. G

1. **Gemini Output**

The treatment landscape for multiple myeloma (MM) has undergone a revolution with the introduction of novel agents. The combination of daratumumab, lenalidomide, bortezomib, and dexamethasone (D-VRd) represents a significant step in this evolution for newly diagnosed patients.

Initially, lenalidomide and dexamethasone (Rd) emerged as a successful therapy for relapsed/refractory MM [4]. Building on this, researchers explored the addition of bortezomib (VRd) to Rd, demonstrating improved progression-free survival in relapsed/refractory patients [2]. This established VRd as a potent frontline therapy for newly diagnosed MM.

The story takes a new turn with daratumumab, a first-in-class CD38-targeted monoclonal antibody. Studies like POLLUX showcased daratumumab's efficacy when combined with lenalidomide and dexamethasone (D-Rd) for relapsed/refractory MM, achieving deeper remissions [4]. This paved the way for the GRIFFIN trial which investigated D-VRd in transplant-eligible, newly diagnosed MM patients [1].

GRIFFIN's findings were groundbreaking. D-VRd significantly extended progression-free survival compared to VRd alone [1]. This established D-VRd as a promising frontline therapy, potentially improving outcomes for newly diagnosed patients. Further research is ongoing to optimize treatment duration and explore potential benefits of stopping daratumumab after achieving minimal residual disease negativity [3, 5].

The journey of D-VRd highlights the continuous advancement in MM treatment. From effective therapies for relapsed disease to a potentially transformative frontline regimen for newly diagnosed patients, this combination therapy offers a beacon of hope for those battling MM.

1. **Llama 2 Output**

**Synopsis: Development and Evolution of Therapy with Daratumumab, Lenalidomide, Bortezomib, and Dexamethasone for Newly Diagnosed Multiple Myeloma**

The treatment of newly diagnosed multiple myeloma (NDMM) has undergone significant evolution with the introduction of novel agents and combination regimens. One such regimen is the quadruplet combination of daratumumab, lenalidomide, bortezomib, and dexamethasone (DRd).

**Early Developments**

- Lenalidomide and dexamethasone (Rd) was established as a standard of care for NDMM [1].
- Bortezomib, a proteasome inhibitor, was added to Rd, forming the triplet combination of VRd, which demonstrated improved response rates and progression-free survival (PFS) [2].

**Introduction of Daratumumab**

- Daratumumab, a monoclonal antibody targeting CD38, was approved for relapsed/refractory multiple myeloma (RRMM) in 2015 [3].
- The phase 3 MAIA trial investigated DRd versus Rd in NDMM, showing significant improvements in PFS and overall response rate (ORR) with the addition of daratumumab [4].

**Evolution of the DRd Regimen**

- The CASSIOPEIA trial compared DRd to VRd in NDMM, demonstrating improved PFS and ORR with DRd [5].
- The GRIFFIN trial investigated DRd with and without maintenance therapy, showing improved PFS with continuous lenalidomide and daratumumab [6].

**Current Status and Future Directions**

- DRd has become a standard of care for NDMM, offering improved efficacy and tolerability [7].
- Ongoing trials are exploring optimal dosing, maintenance strategies, and combinations with other novel agents to further enhance outcomes.

References:

[1] Rajkumar et al. (2014). Lenalidomide and dexamethasone in newly diagnosed multiple myeloma. *New England Journal of Medicine*, 371(10), 906-917.

[2] Durie et al. (2017). Bortezomib, lenalidomide, and dexamethasone in newly diagnosed multiple myeloma. *Blood*, 130(12), 1411-1419.

[3] Lokhorst et al. (2015). Daratumumab, lenalidomide, and dexamethasone for relapsed or refractory multiple myeloma. *New England Journal of Medicine*, 373(15), 1431-1441.

[4] Facon et al. (2019). Daratumumab plus lenalidomide and dexamethasone for newly diagnosed multiple myeloma. *New England Journal of Medicine*, 380(22), 2104-2115.

[5] Moreau et al. (2020). Daratumumab, lenalidomide, and dexamethasone versus lenalidomide and dexamethasone in newly diagnosed multiple myeloma. *Blood*, 135(11), 833-843.

[6] Voorhees et al. (2022). Daratumumab, lenalidomide, and dexamethasone with or without continuous lenalidomide and daratumumab maintenance in newly diagnosed multiple myeloma. *Journal of Clinical Oncology*, 40(10), 1081-1091.

[7] Kumar et al. (2022). Multiple myeloma: diagnosis and treatment. *Mayo Clinic Proceedings*, 97(5), 851-874.

## Carfilzomib, Lenalidomide, and Dexamethasone (KRd)

- 1. **Claude 3.5 Output**

Here's a synopsis of the development and evolution of therapy using carfilzomib, lenalidomide, and dexamethasone (KRd) for newly diagnosed multiple myeloma (NDMM):

The combination of carfilzomib, lenalidomide, and dexamethasone (KRd) emerged as a potent regimen for NDMM, building upon the success of earlier proteasome inhibitor-based combinations.

Carfilzomib, a second-generation proteasome inhibitor, was initially approved for relapsed/refractory multiple myeloma based on its improved efficacy and safety profile compared to first-generation proteasome inhibitors like bortezomib [1].

The development of KRd for NDMM was driven by the need for more effective induction regimens that could achieve deeper and more durable responses. Early phase 1/2 studies demonstrated the feasibility and promising efficacy of this combination in NDMM patients [2].

The ASPIRE trial, although focused on relapsed multiple myeloma, provided important evidence for the efficacy of KRd, showing improved progression-free survival (PFS) compared to lenalidomide and dexamethasone alone [3]. This success in the relapsed setting paved the way for investigations in NDMM.

A pivotal phase 2 study by Jakubowiak et al. evaluated KRd with or without autologous stem cell transplantation (ASCT) in NDMM. The study reported high rates of stringent complete response and minimal residual disease (MRD) negativity, suggesting that KRd could potentially serve as an alternative to ASCT in some patients [4].

The phase 3 ENDURANCE trial compared KRd to bortezomib, lenalidomide, and dexamethasone (VRd) in NDMM patients not intended for immediate ASCT. While the study did not show a PFS benefit for KRd over VRd, it established KRd as an effective alternative, particularly in patients who might not tolerate bortezomib [5].

For transplant-eligible patients, the FORTE trial investigated KRd with or without ASCT, followed by KR or R maintenance. The study demonstrated superior PFS with KRd plus ASCT followed by KR maintenance compared to other arms, highlighting the potential of this approach in high-risk patients [6].

Efforts to optimize the KRd regimen have included investigations into modified dosing schedules to improve tolerability while maintaining efficacy. The CHAMPION-1 trial explored once-weekly dosing of carfilzomib in combination with lenalidomide and dexamethasone, showing promising results [7].

Ongoing research is focusing on identifying specific patient subgroups who may benefit most from KRd, such as those with high-risk cytogenetics. Additionally, studies are exploring the integration of KRd with other novel agents like monoclonal antibodies to further improve outcomes.

The evolution of KRd therapy has also included its evaluation in maintenance settings. The COBRA study is investigating carfilzomib-lenalidomide versus lenalidomide alone as post-transplant maintenance therapy [8].

In conclusion, the development of KRd for NDMM represents a significant advance in myeloma therapy, offering deep and durable responses. Its evolution continues as researchers refine its use, optimize treatment duration, and explore its role in various treatment strategies across the myeloma continuum

- 1. **ChatGPT 4.0 Output**

**Introduction**

The treatment landscape for multiple myeloma has seen significant advancements with the introduction of novel therapeutic agents and combination regimens. Carfilzomib, a second-generation proteasome inhibitor, in combination with lenalidomide and dexamethasone (KRd), has emerged as a highly effective treatment for newly diagnosed multiple myeloma (NDMM). This synopsis outlines the development, clinical trials, and evolution of the KRd regimen.

**Development of Key Therapies**

1. **Carfilzomib (K)**

• **Mechanism of Action**: Carfilzomib selectively and irreversibly inhibits the chymotrypsin-like activity of the proteasome, leading to an accumulation of ubiquitinated proteins, induction of apoptosis, and inhibition of cell proliferation in myeloma cells.

• **Clinical Trials**: Carfilzomib was initially evaluated in relapsed and refractory multiple myeloma (RRMM) patients, showing significant efficacy and improved outcomes compared to traditional therapies (Stewart et al., 2015).

2. **Lenalidomide (R)**

• **Mechanism of Action**: Lenalidomide, an immunomodulatory drug, enhances immune function, inhibits angiogenesis, and directly induces apoptosis in myeloma cells.

• **Clinical Trials**: Lenalidomide combined with dexamethasone (Rd) has been a standard treatment for NDMM, demonstrating improved survival rates (Weber et al., 2007).

3. **Dexamethasone (d)**

• **Mechanism of Action**: Dexamethasone is a corticosteroid that induces apoptosis in myeloma cells and has anti-inflammatory properties.

• **Clinical Trials**: As a part of combination regimens, dexamethasone enhances the therapeutic effects of other agents and helps manage side effects.

**Evolution of Combination Therapy**

1. **Carfilzomib, Lenalidomide, and Dexamethasone (KRd)**

• **Clinical Trials**: The combination of KRd has been extensively studied in multiple clinical trials, establishing its efficacy and safety in NDMM.

2. **Key Clinical Trials**

• **ASPIRE Trial**: The ASPIRE trial was a pivotal phase 3 study comparing KRd to Rd in RRMM patients, demonstrating a significant improvement in progression-free survival (PFS) and overall response rates (ORR) (Stewart et al., 2015). Although the trial focused on RRMM, the promising results laid the groundwork for evaluating KRd in NDMM.

• **ENDURANCE Trial (E1A11)**: This phase 3 trial specifically evaluated KRd versus VRd (bortezomib, lenalidomide, and dexamethasone) in NDMM patients. While KRd showed comparable efficacy to VRd, it provided an alternative regimen for patients with different risk profiles and tolerance levels (Korde et al., 2020).

3. **Real-World Data and Practice**

• **Use in Clinical Practice**: Real-world data has supported the use of KRd in NDMM, highlighting its efficacy and manageable safety profile (Jakubowiak et al., 2012). This regimen has become a viable option for patients, particularly those who may not tolerate bortezomib or who prefer a different side effect profile.

**Conclusion**

The introduction and evolution of the KRd regimen have significantly advanced the treatment of newly diagnosed multiple myeloma. Carfilzomib, combined with lenalidomide and dexamethasone, offers a potent therapeutic option with robust clinical trial support and real-world efficacy. Ongoing research and future studies will continue to refine its use, optimize dosing, and further improve patient outcomes.

**References:**

1. Stewart, A. K., Rajkumar, S. V., Dimopoulos, M. A., et al. (2015). Carfilzomib, lenalidomide, and dexamethasone for relapsed multiple myeloma. *New England Journal of Medicine*, 372(2), 142-152.

2. Weber, D. M., Chen, C., Niesvizky, R., et al. (2007). Lenalidomide plus dexamethasone for relapsed multiple myeloma in North America. *New England Journal of Medicine*, 357(21), 2133-2142.

3. Korde, N., Roschewski, M., Zingone, A., et al. (2020). Treatment with carfilzomib-lenalidomide-dexamethasone (KRd) improves outcomes for patients with newly diagnosed multiple myeloma. *Journal of Clinical Oncology*, 38(32), 3849-3860.

4. Jakubowiak, A. J., Dytfeld, D., Griffith, K. A., et al. (2012). A phase 1/2 study of carfilzomib in combination with lenalidomide and low-dose dexamethasone as a frontline treatment for multiple myeloma. *Blood*, 120(9), 1801-1809.

This synopsis provides a detailed overview of the development and clinical evolution of the KRd regimen, supported by key clinical trial data and literature references.

- 1. **Gemini Output**

**Carfilzomib, Lenalidomide, and Dexamethasone: A Triple Threat for Newly Diagnosed Multiple Myeloma**

The fight against multiple myeloma (MM) has seen significant progress with the introduction of carfilzomib, lenalidomide, and dexamethasone (KRd). This powerful combination therapy has become a cornerstone in the treatment of newly diagnosed MM.

Prior to KRd, lenalidomide and dexamethasone (Rd) established itself as a successful treatment for relapsed/refractory MM [2]. Researchers then investigated the addition of carfilzomib, a proteasome inhibitor, to Rd. Clinical trials like the FOCUS trial demonstrated that KRd significantly improved progression-free survival compared to Rd alone in patients with relapsed MM [2]. These findings fueled interest in exploring KRd's potential for newly diagnosed patients.

The pivotal study for KRd in this setting was the CARRTERA trial. It compared KRd induction/consolidation followed by autologous stem cell transplantation (ASCT) to a traditional treatment regimen (melphalan, prednisone, and bortezomib followed by ASCT) in transplant-eligible patients with newly diagnosed MM [1]. CARRTERA's results were impressive, showing KRd led to deeper remissions and potentially improved overall survival compared to the control arm [1].

Further research on KRd continues to refine its use in newly diagnosed MM. Studies like the ELARA trial are exploring the efficacy of KRd with and without elotuzumab, another antibody therapy, to determine the optimal treatment approach [3].

The development of KRd exemplifies the ongoing pursuit of better outcomes in MM. From demonstrating effectiveness in relapsed disease to becoming a cornerstone of treatment for newly diagnosed patients, KRd offers a powerful option for those battling this challenging condition.

- 1. **Llama 2 Output**

**Synopsis: Development and Evolution of Therapy with Carfilzomib, Lenalidomide, and Dexamethasone for Newly Diagnosed Multiple Myeloma**

The treatment of newly diagnosed multiple myeloma (NDMM) has undergone significant advancements with the introduction of novel proteasome inhibitors and combination regimens. One such regimen is the triplet combination of carfilzomib, lenalidomide, and dexamethasone (KRd).

**Early Developments**

- Lenalidomide and dexamethasone (Rd) was established as a standard of care for NDMM [1].
- Carfilzomib, a second-generation proteasome inhibitor, was approved for relapsed/refractory multiple myeloma (RRMM) in 2012 [2].

**Introduction of KRd**

- The phase 1/2 study of KRd in NDMM demonstrated promising efficacy and tolerability [3].
- The phase 3 ASPIRE trial compared KRd to Rd in RRMM, showing significant improvements in progression-free survival (PFS) and overall response rate (ORR) with the addition of carfilzomib [4].

**Evolution of the KRd Regimen**

- The phase 3 ENDURANCE trial compared KRd to bortezomib, lenalidomide, and dexamethasone (VRd) in NDMM, demonstrating similar efficacy but improved tolerability with KRd [5].
- The phase 2 FORTE trial investigated KRd with different carfilzomib dosing schedules, showing improved PFS with the 56 mg/m² dose [6].

**Current Status and Future Directions**

- KRd has become a standard of care for NDMM, offering improved efficacy and tolerability [7].
- Ongoing trials are exploring optimal dosing, maintenance strategies, and combinations with other novel agents to further enhance outcomes.

References:

[1] Rajkumar et al. (2014). Lenalidomide and dexamethasone in newly diagnosed multiple myeloma. *New England Journal of Medicine*, 371(10), 906-917.

[2] Siegel et al. (2012). Carfilzomib in relapsed or refractory multiple myeloma. *Blood*, 120(14), 2811-2818.

[3] Wang et al. (2013). Carfilzomib, lenalidomide, and dexamethasone in newly diagnosed multiple myeloma. *Blood*, 122(21), 2326-2333.

[4] Stewart et al. (2015). Carfilzomib, lenalidomide, and dexamethasone for relapsed or refractory multiple myeloma. *New England Journal of Medicine*, 373(15), 1426-1437.

[5] Kumar et al. (2019). Carfilzomib, lenalidomide, and dexamethasone versus bortezomib, lenalidomide, and dexamethasone in newly diagnosed multiple myeloma. *Blood*, 133(11), 939-948.

[6] Gay et al. (2020). Carfilzomib, lenalidomide, and dexamethasone in newly diagnosed multiple myeloma: the FORTE trial. *Journal of Clinical Oncology*, 38(15), 1704-1714.

[7] Kumar et al. (2022). Multiple myeloma: diagnosis and treatment. *Mayo Clinic Proceedings*, 97(5), 851-874.

## VTD-PACE

- 1. **Claude 3.5 Output**

Here's a synopsis of the development and evolution of VTD-PACE (bortezomib, thalidomide, dexamethasone, cisplatin, doxorubicin, cyclophosphamide, and etoposide) therapy for multiple myeloma:

VTD-PACE is an intensive, multi-agent chemotherapy regimen developed for the treatment of aggressive or advanced multiple myeloma, particularly in cases of relapsed/refractory disease or high-risk newly diagnosed patients.

The evolution of VTD-PACE builds upon earlier intensive regimens, notably DT-PACE (dexamethasone, thalidomide, cisplatin, doxorubicin, cyclophosphamide, and etoposide), which was developed by Barlogie and colleagues at the University of Arkansas for Medical Sciences [1].

DT-PACE was initially used as part of the Total Therapy protocols, aimed at achieving long-term remission or potential cure in newly diagnosed multiple myeloma patients. The addition of bortezomib to create VTD-PACE was a natural progression following the success of bortezomib in multiple myeloma treatment [2].

The rationale behind VTD-PACE is to combine agents with different mechanisms of action to overcome drug resistance and achieve rapid disease control. Bortezomib (proteasome inhibitor), thalidomide (immunomodulator), and dexamethasone (corticosteroid) form the backbone, while the traditional chemotherapy agents target rapidly dividing cells [3].

Early studies of VTD-PACE showed promising results in relapsed/refractory multiple myeloma. A retrospective analysis by Lakshman et al. reported an overall response rate of 83% in heavily pretreated patients, with a median progression-free survival of 5.5 months [4].

VTD-PACE has been incorporated into various treatment strategies:

1. As induction therapy for high-risk newly diagnosed patients: In the Total Therapy 3 protocol, VTD-PACE was used as part of an intensive approach, resulting in improved outcomes for high-risk patients compared to historical controls [5].
2. As salvage therapy for relapsed/refractory disease: Several studies have demonstrated the efficacy of VTD-PACE in this setting, particularly as a bridge to autologous stem cell transplantation (ASCT) [6].
3. As part of conditioning regimens: Modified versions of VTD-PACE have been explored as conditioning regimens before ASCT, aiming to deepen response prior to transplant [7].

The toxicity profile of VTD-PACE is significant, including myelosuppression, infections, and thromboembolic events. This has led to efforts to optimize the regimen:

1. Dose modifications: Various studies have explored reduced doses or altered schedules to improve tolerability while maintaining efficacy [8].
2. Supportive care: The use of growth factors, prophylactic antibiotics, and anticoagulation has become standard to manage side effects [9].
3. Patient selection: Given its intensity, careful patient selection based on performance status and comorbidities is crucial [10].

Recent developments in VTD-PACE therapy include:

1. Incorporation of newer agents: Some centers have explored replacing thalidomide with lenalidomide or adding daratumumab to further enhance efficacy [11].
2. Outpatient administration: Efforts to administer modified versions of VTD-PACE in outpatient settings to improve patient quality of life and reduce hospitalization costs [12].
3. Biomarker-guided therapy: Ongoing research aims to identify predictive biomarkers to better select patients who would benefit most from this intensive approach [13].

In conclusion, VTD-PACE represents an important treatment option in the multiple myeloma armamentarium, particularly for aggressive disease. Its evolution continues as researchers refine its use, explore combinations with newer agents, and work to optimize its risk-benefit profile

- 1. **ChatGPT 4.0 Output**

**Synopsis for the Development and Evolution of Therapy with VTD-PACE for Multiple Myeloma**

**Introduction**

VTD-PACE (bortezomib, thalidomide, dexamethasone, cisplatin, doxorubicin, cyclophosphamide, and etoposide) is a multi-agent chemotherapy regimen used in the treatment of multiple myeloma, particularly in high-risk or refractory cases. This regimen combines proteasome inhibition, immunomodulation, and traditional cytotoxic chemotherapy to achieve synergistic effects. This synopsis outlines the development, clinical trials, and evolution of VTD-PACE in the management of multiple myeloma.

**Development of Key Therapies**

1. **Bortezomib (V)**

• **Mechanism of Action**: Bortezomib is a proteasome inhibitor that disrupts the ubiquitin-proteasome pathway, leading to the accumulation of misfolded proteins and apoptosis in myeloma cells.

• **Clinical Trials**: Bortezomib has been a cornerstone in multiple myeloma treatment, showing efficacy as a single agent and in combination regimens (Richardson et al., 2003).

2. **Thalidomide (T)**

• **Mechanism of Action**: Thalidomide is an immunomodulatory drug that inhibits angiogenesis and modulates the immune response, enhancing the cytotoxic effects against myeloma cells.

• **Clinical Trials**: Thalidomide has been effective in combination with dexamethasone and other agents for multiple myeloma (Singhal et al., 1999).

3. **Dexamethasone (D)**

• **Mechanism of Action**: Dexamethasone is a corticosteroid that induces apoptosis in lymphoid cells, including myeloma cells.

• **Clinical Trials**: Dexamethasone is widely used in combination regimens to potentiate the effects of other therapeutic agents (Rajkumar et al., 2005).

4. **PACE Components**

• **Cisplatin (P)**: A platinum-based chemotherapy agent that causes DNA crosslinking and apoptosis.

• **Doxorubicin (A)**: An anthracycline antibiotic that intercalates DNA and inhibits topoisomerase II.

• **Cyclophosphamide (C)**: An alkylating agent that forms DNA crosslinks and induces apoptosis.

• **Etoposide (E)**: A topoisomerase II inhibitor that prevents DNA re-ligation, leading to DNA strand breaks and apoptosis.

**Evolution of Combination Therapy**

1. **VTD-PACE Regimen**

• **Clinical Rationale**: The combination of VTD-PACE leverages the different mechanisms of action to overcome drug resistance and achieve deeper responses in high-risk and refractory multiple myeloma patients.

• **Efficacy and Safety**: The regimen has been shown to be effective in inducing high response rates, including in patients who have relapsed after other treatments (Kumar et al., 2011).

2. **Key Clinical Trials**

• **Initial Studies**: Early studies of VTD-PACE demonstrated significant activity in relapsed and refractory multiple myeloma, with notable response rates and acceptable safety profiles (Kumar et al., 2011; Reece et al., 2009).

• **Subsequent Trials**: Further trials have refined the regimen, optimizing dosing schedules and supportive care to enhance tolerability and efficacy (Shah et al., 2015).

3. **Real-World Data and Practice**

• **Use in Clinical Practice**: Real-world experiences have corroborated the clinical trial findings, supporting the use of VTD-PACE in patients with aggressive disease or those who have exhausted other treatment options (Kumar et al., 2011).

**Conclusion**

The VTD-PACE regimen represents a powerful therapeutic option for multiple myeloma, particularly for high-risk or refractory cases. By combining agents with diverse mechanisms of action, VTD-PACE achieves substantial anti-myeloma activity, offering hope for patients with challenging disease profiles. Ongoing research aims to further optimize this regimen, balancing efficacy with tolerability to improve patient outcomes.

**References:**

1. Richardson, P. G., Barlogie, B., Berenson, J., et al. (2003). A phase 2 study of bortezomib in relapsed, refractory myeloma. *New England Journal of Medicine*, 348(26), 2609-2617.

2. Singhal, S., Mehta, J., Desikan, R., et al. (1999). Antitumor activity of thalidomide in refractory multiple myeloma. *New England Journal of Medicine*, 341(21), 1565-1571.

3. Rajkumar, S. V., Jacobus, S., Callander, N. S., et al. (2005). Lenalidomide plus high-dose dexamethasone versus dexamethasone alone in newly diagnosed multiple myeloma: a randomized trial. *Lancet Oncology*, 6(1), 29-37.

4. Kumar, S., Reeder, C. B., Lacy, M. Q., et al. (2011). Impact of lenalidomide therapy on the risk of thromboembolic events in newly diagnosed multiple myeloma: a single-institution experience. *Mayo Clinic Proceedings*, 86(16), 1598-1606.

5. Reece, D. E., Zeldis, J. B., Yoon, S. S., et al. (2009). Lenalidomide (Revlimid) plus dexamethasone followed by autologous stem cell transplant in newly diagnosed multiple myeloma: A multicenter phase II study. *Blood*, 114(22), 2513.

6. Shah, J. J., Stadtmauer, E. A., Abonour, R., et al. (2015). Carfilzomib, lenalidomide, and dexamethasone (CRd) as front-line therapy for multiple myeloma: A phase 1/2 study. *Blood*, 126(21), 1842-1848.

- 1. **Gemini Output**

**VTD-PACE: An Intensive Regimen for Relapsed/Refractory Multiple Myeloma**

VTD-PACE, a multi-drug chemotherapy regimen, has emerged as a powerful tool for managing relapsed/refractory multiple myeloma (MM). While not typically used for newly diagnosed patients, VTD-PACE plays a crucial role in addressing advanced MM.

The components of VTD-PACE – bortezomib (V), thalidomide (T), dexamethasone (d), cisplatin (P), doxorubicin (A), cyclophosphamide (C), and etoposide (E) – represent a potent combination targeting MM cells through various mechanisms. The regimen was developed as part of "total therapy" protocols, aiming for disease control before stem cell transplantation [1].

Initial studies focused on VTD-PACE's efficacy in the relapsed setting. A study by Kumar et al. demonstrated promising results, with patients achieving significant responses and some proceeding to successful stem cell transplants [4]. VTD-PACE's effectiveness has been further confirmed in other studies, highlighting its potential to improve outcomes in relapsed MM [2, 3].

It's important to note that VTD-PACE is an intensive regimen with a significant side effect profile. Studies report that infections are a common complication, requiring close monitoring and supportive care [2, 3]. Due to its intensity, VTD-PACE is often used as a bridge therapy to prepare patients for subsequent treatments like stem cell transplant or newer targeted therapies.

While VTD-PACE is not currently a first-line therapy for newly diagnosed MM, its role in managing relapsed/refractory disease remains valuable. Ongoing research might explore ways to optimize VTD-PACE or VTD-PACE-like regimens to improve tolerability and potentially expand their use in specific patient populations.

In conclusion, VTD-PACE represents a significant advancement in treating relapsed/refractory MM. This intensive regimen offers a chance for disease control and potentially paves the way for further treatments, offering hope for patients battling advanced MM.

Llama

**Synopsis: Development and Evolution of Therapy with VTD-PACE for Multiple Myeloma**

VTD-PACE, a combination of bortezomib, thalidomide, dexamethasone, cisplatin, doxorubicin, cyclophosphamide, and etoposide, has emerged as a potent regimen for multiple myeloma (MM) treatment.

**Early Developments**

- VTD (bortezomib, thalidomide, and dexamethasone) demonstrated significant efficacy in relapsed/refractory MM [1].
- PACE (cisplatin, doxorubicin, cyclophosphamide, and etoposide) was used in aggressive MM cases [2].

**Introduction of VTD-PACE**

- The phase 2 study by Reeder et al. (2014) combined VTD with PACE, showing improved response rates and survival in relapsed/refractory MM [3].
- The phase 2 study by Rodriguez-Otero et al. (2015) confirmed VTD-PACE's efficacy in heavily pretreated MM patients [4].

**Evolution and Optimization**

- The phase 2 study by Niesvizky et al. (2017) modified VTD-PACE by substituting liposomal doxorubicin for doxorubicin, reducing toxicity while maintaining efficacy [5].
- The phase 2 study by Chari et al. (2020) explored VTD-PACE with and without maintenance therapy, showing improved progression-free survival with maintenance [6].

**Current Status and Future Directions**

- VTD-PACE remains a viable option for heavily pretreated or aggressive MM cases [7].
- Ongoing trials are investigating VTD-PACE in combination with novel agents, such as monoclonal antibodies, to further enhance outcomes.

References:

[1] Ciolli et al. (2013). Bortezomib, thalidomide, and dexamethasone in relapsed/refractory multiple myeloma. *Blood*, 122(14), 2315-2322.

[2] Kumar et al. (2008). Aggressive multiple myeloma: treatment with PACE. *Leukemia*, 22(10), 1939-1945.

[3] Reeder et al. (2014). VTD-PACE: a novel combination for relapsed/refractory multiple myeloma. *Blood*, 124(11), 1737-1744.

[4] Rodriguez-Otero et al. (2015). VTD-PACE in heavily pretreated multiple myeloma patients. *European Journal of Haematology*, 95(3), 251-258.

[5] Niesvizky et al. (2017). Modified VTD-PACE with liposomal doxorubicin in relapsed/refractory multiple myeloma. *Journal of Clinical Oncology*, 35(22), 2444-2451.

[6] Chari et al. (2020). VTD-PACE with and without maintenance therapy in relapsed/refractory multiple myeloma. *Blood*, 135(11), 833-843.

[7] Kumar et al. (2022). Multiple myeloma: diagnosis and treatment. *Mayo Clinic Proceedings*, 97(5), 851-874.

- 1. **Llama 2 Output**

Synopsis: Development and Evolution of Therapy with VTD-PACE for Multiple Myeloma

VTD-PACE, a combination of bortezomib, thalidomide, dexamethasone, cisplatin, doxorubicin,

cyclophosphamide, and etoposide, has emerged as a potent regimen for multiple myeloma

(MM) treatment.

Early Developments

• VTD (bortezomib, thalidomide, and dexamethasone) demonstrated significant efficacy in

relapsed/refractory MM [1].

• PACE (cisplatin, doxorubicin, cyclophosphamide, and etoposide) was used in aggressive MM

cases [2].

Introduction of VTD-PACE

• The phase 2 study by Reeder et al. (2014) combined VTD with PACE, showing improved

response rates and survival in relapsed/refractory MM [3].

• The phase 2 study by Rodriguez-Otero et al. (2015) confirmed VTD-PACE's efficacy in heavily

pretreated MM patients [4].

Evolution and Optimization

• The phase 2 study by Niesvizky et al. (2017) modified VTD-PACE by substituting liposomal

doxorubicin for doxorubicin, reducing toxicity while maintaining efficacy [5].

• The phase 2 study by Chari et al. (2020) explored VTD-PACE with and without maintenance

therapy, showing improved progression-free survival with maintenance [6].

Current Status and Future Directions

• VTD-PACE remains a viable option for heavily pretreated or aggressive MM cases [7].

• Ongoing trials are investigating VTD-PACE in combination with novel agents, such as

monoclonal antibodies, to further enhance outcomes.

References:

[1] Ciolli et al. (2013). Bortezomib, thalidomide, and dexamethasone in relapsed/refractory

multiple myeloma. Blood, 122(14), 2315-2322.

[2] Kumar et al. (2008). Aggressive multiple myeloma: treatment with PACE. Leukemia,

22(10), 1939-1945.

[3] Reeder et al. (2014). VTD-PACE: a novel combination for relapsed/refractory multiple

myeloma. Blood, 124(11), 1737-1744.

[4] Rodriguez-Otero et al. (2015). VTD-PACE in heavily pretreated multiple myeloma patients.

European Journal of Haematology, 95(3), 251-258.

[5] Niesvizky et al. (2017). Modified VTD-PACE with liposomal doxorubicin in

relapsed/refractory multiple myeloma. Journal of Clinical Oncology, 35(22), 2444-2451.

[6] Chari et al. (2020). VTD-PACE with and without maintenance therapy in relapsed/refractory

multiple myeloma. Blood, 135(11), 833-843.

[7] Kumar et al. (2022). Multiple myeloma: diagnosis and treatment. Mayo Clinic Proceedings,

97(5), 851-874.

## Dara-VCD

- 1. **Claude 3.5 Output**

Here's a synopsis of the development and evolution of DaraVCD (Daratumumab, Bortezomib, Cyclophosphamide, and Dexamethasone) therapy for AL Amyloidosis:

AL Amyloidosis is a rare plasma cell dyscrasia characterized by the deposition of misfolded light chain proteins in various organs. Treatment strategies have evolved from those used in multiple myeloma, given the similar underlying pathology.

The development of DaraVCD for AL Amyloidosis builds upon the success of VCD (Bortezomib, Cyclophosphamide, and Dexamethasone) regimen, which became a standard of care for AL Amyloidosis based on its rapid and deep hematologic responses [1].

Daratumumab, a monoclonal antibody targeting CD38, was initially approved for multiple myeloma. Its efficacy in plasma cell disorders led to investigations in AL Amyloidosis. The rationale for adding daratumumab to VCD was to enhance the depth and speed of hematologic responses, which are crucial for organ improvement in AL Amyloidosis [2].

The evolution of DaraVCD therapy can be traced through several key studies:

1. Early case reports and small series demonstrated the efficacy of daratumumab in relapsed/refractory AL Amyloidosis, providing a rationale for its use in combination regimens [3].
2. The phase 3 ANDROMEDA trial was a pivotal study that compared DaraVCD to VCD in newly diagnosed AL Amyloidosis. This study showed significantly higher rates of complete hematologic response and improved organ responses with DaraVCD [4].
3. Long-term follow-up data from ANDROMEDA presented at subsequent conferences have continued to show sustained benefits of DaraVCD, including improved progression-free survival and deeper organ responses [5].

The development of DaraVCD has also focused on optimizing its administration:

1. Subcutaneous formulation: Studies have explored the use of subcutaneous daratumumab to improve patient convenience and reduce infusion-related reactions [6].
2. Modified dosing schedules: Efforts to optimize the frequency and duration of daratumumab administration are ongoing, aiming to balance efficacy and tolerability [7].
3. Risk-adapted approaches: Research is focusing on tailoring treatment intensity based on patient-specific factors such as organ involvement and disease severity [8].

The success of DaraVCD has led to its rapid adoption in clinical practice:

1. Guidelines update: Major hematology societies have updated their guidelines to include DaraVCD as a preferred first-line option for AL Amyloidosis [9].
2. Real-world evidence: Studies reporting real-world outcomes with DaraVCD are emerging, generally confirming the efficacy seen in clinical trials [10].

Ongoing research is addressing several key areas:

1. Biomarkers: Efforts to identify predictive biomarkers for response to DaraVCD and for early detection of organ response [11].
2. Maintenance therapy: Investigating the role of daratumumab maintenance after initial therapy to prolong remission [12].
3. Combination with other novel agents: Exploring the addition of other targeted therapies to further improve outcomes [13].
4. Treatment sequencing: Determining optimal treatment sequences, including the role of DaraVCD in relapsed/refractory settings [14].

In conclusion, the development of DaraVCD represents a significant advance in the treatment of AL Amyloidosis, offering improved depth and speed of hematologic responses, which translate into better organ outcomes. Its evolution continues as researchers refine its use, explore long-term outcomes, and investigate ways to further improve patient care in this challenging disease.

1. **ChatGPT 4.0 Output**

### Synopsis for the Development and Evolution of Therapy with DaraVCD for AL Amyloidosis

#### Introduction

AL amyloidosis is a rare and often fatal disorder characterized by the deposition of misfolded light chain proteins in various tissues and organs. Effective treatment regimens aim to suppress the underlying plasma cell dyscrasia and promote organ response. The combination therapy of daratumumab, bortezomib, cyclophosphamide, and dexamethasone (DaraVCD) has emerged as a promising treatment approach for patients with AL amyloidosis. This synopsis outlines the development, clinical trials, and evolution of DaraVCD therapy.

#### Development of Key Therapies

1. **Daratumumab (Dara)**

- **Mechanism of Action**: Daratumumab is a human monoclonal antibody targeting CD38, expressed on plasma cells. It induces cell death through complement-dependent cytotoxicity (CDC), antibody-dependent cellular cytotoxicity (ADCC), and antibody-dependent cellular phagocytosis (ADCP).

- **Clinical Trials**: Daratumumab has shown efficacy in treating multiple myeloma, leading to its investigation in AL amyloidosis due to its ability to target abnormal plasma cells (Plesner et al., 2020).

2. **Bortezomib (V)**

- **Mechanism of Action**: Bortezomib is a proteasome inhibitor that induces apoptosis in plasma cells by disrupting protein homeostasis.

- **Clinical Trials**: Bortezomib, combined with cyclophosphamide and dexamethasone (VCD), has been a cornerstone in the treatment of AL amyloidosis, demonstrating significant hematologic response rates (Palladini et al., 2012).

3. **Cyclophosphamide (C)**

- **Mechanism of Action**: Cyclophosphamide is an alkylating agent that interferes with DNA replication, leading to apoptosis of rapidly dividing cells, including abnormal plasma cells.

- **Clinical Trials**: Cyclophosphamide has been effectively used in combination regimens for AL amyloidosis to enhance the cytotoxic effects on plasma cells (Palladini et al., 2012).

4. **Dexamethasone (d)**

- **Mechanism of Action**: Dexamethasone is a corticosteroid that induces apoptosis in lymphoid cells, including plasma cells, and helps mitigate inflammation and treatment-related side effects.

- **Clinical Trials**: As part of combination regimens, dexamethasone potentiates the efficacy of other agents and aids in reducing treatment-related symptoms (Kastritis et al., 2020).

#### Evolution of Combination Therapy

1. **DaraVCD Regimen**

- **Clinical Rationale**: Combining daratumumab with the VCD regimen aims to leverage the potent anti-plasma cell effects of daratumumab and the established efficacy of VCD, resulting in a more effective treatment for AL amyloidosis.

- **Efficacy and Safety**: The regimen has been shown to induce deep hematologic responses and organ improvements, with an acceptable safety profile (Kastritis et al., 2020).

2. **Key Clinical Trials**

- **ANDROMEDA Trial**: The phase 3 ANDROMEDA trial evaluated the addition of daratumumab to VCD (DaraVCD) versus VCD alone in newly diagnosed AL amyloidosis patients. The trial demonstrated superior hematologic complete response rates and improved organ responses in the DaraVCD group compared to VCD alone (Kastritis et al., 2020).

- **Phase 2 Studies**: Earlier phase 2 studies supported the safety and efficacy of daratumumab in combination with VCD, providing the foundation for the ANDROMEDA trial (Sanchorawala et al., 2020).

3. **Real-World Data and Practice**

- **Use in Clinical Practice**: Real-world data has reinforced the clinical trial findings, with reports of high response rates and manageable toxicity in patients treated with DaraVCD (Vaxman et al., 2021).

#### Conclusion

The development and evolution of the DaraVCD regimen have significantly impacted the treatment landscape for AL amyloidosis. The combination of daratumumab with bortezomib, cyclophosphamide, and dexamethasone offers a potent therapeutic option that improves hematologic and organ responses, providing hope for better outcomes in this challenging disease. Ongoing research and long-term follow-up studies will continue to refine the regimen and optimize patient care.

---

**References:**

1. Plesner, T., Arkenau, H. T., Gimsing, P., et al. (2020). Daratumumab in combination with cyclophosphamide, bortezomib, and dexamethasone (CyBorD) in patients with newly diagnosed AL amyloidosis: safety run-in results of ANDROMEDA. *Blood*, 136(1), 71-80.

2. Palladini, G., Kastritis, E., Maurer, M. S., et al. (2012). Bortezomib-based regimens in the treatment of patients with newly diagnosed systemic light-chain (AL) amyloidosis. *Blood*, 119(19), 4391-4400.

3. Kastritis, E., Palladini, G., Minnema, M. C., et al. (2020). Daratumumab-based treatment for immunoglobulin light-chain amyloidosis. *New England Journal of Medicine*, 382(9), 694-704.

4. Sanchorawala, V., Palladini, G., Kukreti, V., et al. (2020). Safety and efficacy of daratumumab in AL amyloidosis: preliminary results from the phase 2 ANDROMEDA study. *Journal of Clinical Oncology*, 38(15_suppl), 8502.

5. Vaxman, I., Gertz, M. A., Kumar, S., et al. (2021). Real-world data of daratumumab-based therapy in AL amyloidosis. *British Journal of Haematology*, 192(1), 102-106.

---

This synopsis provides a detailed overview of the development and clinical evolution of the DaraVCD regimen, supported by key clinical trial data and literature references.

1. **Gemini Output**

DaraVCD: A New Standard for AL Amyloidosis

The treatment landscape for AL amyloidosis has undergone a significant transformation with the advent of DaraVCD – a combination of daratumumab, bortezomib, cyclophosphamide, and dexamethasone.

Prior to DaraVCD, treatment for AL amyloidosis primarily relied on chemotherapy regimens with variable efficacy and substantial toxicity. The introduction of daratumumab, a CD38-targeted monoclonal antibody, marked a turning point. Early studies demonstrated its effectiveness in relapsed AL amyloidosis, with significant hematologic response rates [1].

The pivotal ANDROMEDA trial compared DaraVCD to VCD (bortezomib, cyclophosphamide, dexamethasone) in newly diagnosed AL amyloidosis patients. Results demonstrated superior hematologic and organ response rates with DaraVCD, establishing it as a new standard of care for this patient population [2].

Building on this success, ongoing research is exploring the optimal treatment approach for AL amyloidosis. Studies are investigating the role of autologous stem cell transplantation after DaraVCD induction to further improve outcomes [3]. Additionally, the long-term effects of DaraVCD on organ damage and overall survival are being closely monitored.

In summary, DaraVCD represents a major advancement in the treatment of AL amyloidosis. Its efficacy and tolerability profile have significantly improved patient outcomes, offering hope for a better prognosis for individuals with this challenging disease.

Citations:

1. Daratumumab in AL amyloidosis. Blood. 2021;140(22):2317-2327.

2. ANDROMEDA: Dara-VCd for newly diagnosed AL amyloidosis: the next galaxy or a new standard of care? Ther Adv Hematol. 2022;13:2035357.

3. A phase III, randomized study of daratumumab, cyclophosphamide, bortezomib and dexamethasone (DARA-VCD) induction followed by autologous stem cell transplant or DARA-VCD consolidation and daratumumab maintenance in patients with newly diagnosed AL amyloidosis. J Clin Oncol. 2024;42(16 Suppl):TPS7575.

1. **Llama 2 Output**

**Synopsis: Development and Evolution of Therapy with DaraVCD for AL Amyloidosis**

DaraVCD, a combination of daratumumab, bortezomib, cyclophosphamide, and dexamethasone, has emerged as a promising regimen for light-chain (AL) amyloidosis treatment.

**Early Developments**

- Bortezomib-based regimens showed efficacy in AL amyloidosis [1].
- Daratumumab, a monoclonal antibody targeting CD38, demonstrated activity in relapsed/refractory multiple myeloma and AL amyloidosis [2].

**Introduction of DaraVCD**

- The phase 2 ANDROMEDA trial investigated DaraVCD in newly diagnosed AL amyloidosis, showing high overall response rates (ORR) and organ response rates [3].
- The phase 2 CENTAURUS trial confirmed DaraVCD's efficacy in relapsed/refractory AL amyloidosis [4].

**Evolution and Optimization**

- The phase 2 AMETHYST trial explored DaraVCD with and without maintenance therapy, demonstrating improved progression-free survival with maintenance [5].
- Ongoing trials are investigating DaraVCD in combination with other novel agents and exploring optimal dosing and duration.

**Current Status and Future Directions**

- DaraVCD has become a standard of care for AL amyloidosis, offering improved ORR and organ response rates [6].
- Future research will focus on optimizing DaraVCD-based regimens, exploring biomarkers for treatment response, and investigating its use in combination with other therapies.

References:

[1] Reece et al. (2011). Bortezomib in AL amyloidosis. *Leukemia*, 25(11), 1646-1654.

[2] Usmani et al. (2015). Daratumumab in relapsed/refractory multiple myeloma. *New England Journal of Medicine*, 373(15), 1431-1441.

[3] Kastritis et al. (2020). Daratumumab, bortezomib, cyclophosphamide, and dexamethasone in newly diagnosed AL amyloidosis. *Blood*, 135(11), 833-843.

[4] Palladini et al. (2020). Daratumumab, bortezomib, cyclophosphamide, and dexamethasone in relapsed/refractory AL amyloidosis. *Journal of Clinical Oncology*, 38(15), 1704-1714.

[5] Merlini et al. (2022). Daratumumab, bortezomib, cyclophosphamide, and dexamethasone with and without maintenance therapy in AL amyloidosis. *Blood Advances*, 6(2), 338-347.

[6] Falk et al. (2022). AL amyloidosis: diagnosis and treatment. *Blood*, 139(11), 1551-1564.

## Elranatamab

- 1. **Claude 3.5 Output**

Here's a synopsis of the development and evolution of therapy with elranatamab for relapsed multiple myeloma:

Elranatamab is a bispecific antibody targeting B-cell maturation antigen (BCMA) and CD3, developed for the treatment of relapsed/refractory multiple myeloma (RRMM). Its development represents part of a broader trend towards immunotherapy approaches in multiple myeloma treatment.

The rationale for targeting BCMA stems from its near-universal expression on multiple myeloma cells and its role in promoting plasma cell survival. Elranatamab's bispecific design allows it to engage T cells (via CD3) and redirect them to attack BCMA-expressing myeloma cells [1].

Key milestones in the development of elranatamab include:

1. Preclinical studies: Initial in vitro and animal studies demonstrated the potent anti-myeloma activity of elranatamab, providing the basis for clinical development [2].

2. Phase 1 trial: The first-in-human study (MagnetisMM-1) established the safety and preliminary efficacy of elranatamab in heavily pretreated RRMM patients. This study also helped determine the recommended phase 2 dose [3].

3. Phase 2 trials: The MagnetisMM-3 trial evaluated elranatamab in triple-class exposed RRMM patients, showing promising overall response rates and durability of response [4].

4. Pivotal trial: The ongoing MagnetisMM-5 study is comparing elranatamab to standard of care in lenalidomide-refractory multiple myeloma patients [5].

The evolution of elranatamab therapy has focused on several key areas:

1. Dosing optimization: Studies have explored various dosing schedules and step-up dosing approaches to mitigate cytokine release syndrome (CRS) while maintaining efficacy [6].

2. Subcutaneous administration: Unlike some other bispecific antibodies, elranatamab is administered subcutaneously, potentially improving patient convenience [7].

3. Combination strategies: Ongoing trials are investigating elranatamab in combination with other agents, including immunomodulators and proteasome inhibitors [8].

4. Earlier lines of therapy: While initial development focused on heavily pretreated patients, there is growing interest in evaluating elranatamab in earlier lines of therapy [9].

Key findings from clinical trials include:

1. Efficacy: High overall response rates (60-80%) have been reported in heavily pretreated RRMM patients, including those refractory to anti-CD38 antibodies [10].

2. Safety profile: The most common adverse events include CRS and neurologic toxicities, which are generally manageable with supportive care and tend to occur early in treatment [11].

3. Duration of response: Preliminary data suggest durable responses, with median duration of response not reached in some studies at the time of reporting [12].

Ongoing research and future directions include:

1. Biomarker studies: Efforts to identify predictive biomarkers for response and toxicity to elranatamab [13].

2. Resistance mechanisms: Investigations into mechanisms of resistance to BCMA-targeted therapies and strategies to overcome them [14].

3. Comparative studies: Head-to-head comparisons with other BCMA-targeted therapies (e.g., CAR-T cells, antibody-drug conjugates) are of interest to define optimal sequencing strategies [15].

4. Quality of life assessments: Given the potential for long-term treatment, understanding the impact of elranatamab on patient-reported outcomes is crucial [16].

In conclusion, the development of elranatamab represents a significant advance in immunotherapy for RRMM. Its evolution continues as researchers refine its use, explore combination strategies, and investigate its potential in earlier lines of therapy. The promising efficacy and manageable safety profile position elranatamab as an important addition to the multiple myeloma treatment landscape.

Would you like me to elaborate on any specific aspect of this synopsis or provide more information about elranatamab or bispecific antibodies in multiple myeloma?

[Note: As an AI language model, I don't have access to a live database of citations. The references mentioned above are based on my training data and may not reflect the most recent publications. Please verify these citations independently.]

- 1. **ChatGPT 4.0 Output**

### Synopsis for the Development and Evolution of Therapy with Elranatamab for Relapsed Multiple Myeloma

#### Introduction

Elranatamab, a bispecific antibody targeting B-cell maturation antigen (BCMA) and CD3, represents a novel therapeutic approach for relapsed multiple myeloma. By redirecting T-cells to target and eliminate BCMA-expressing myeloma cells, elranatamab has shown promise in early clinical trials. This synopsis outlines the development, clinical trials, and evolution of elranatamab therapy for relapsed multiple myeloma.

#### Development of Key Therapy

1. **Elranatamab (Elra)**

- **Mechanism of Action**: Elranatamab is designed to simultaneously bind to BCMA on multiple myeloma cells and CD3 on T-cells. This dual engagement activates and redirects T-cells to lyse BCMA-expressing myeloma cells, leveraging the body's immune system to combat the disease.

- **Clinical Trials**: Early-phase trials have demonstrated significant anti-myeloma activity and an acceptable safety profile in heavily pretreated patients with relapsed or refractory multiple myeloma (Mateos et al., 2022).

#### Evolution of Therapy

1. **Preclinical Development**

- **Rationale and Design**: Preclinical studies highlighted the potential of targeting BCMA due to its high expression on myeloma cells and limited expression on normal tissues. Elranatamab was engineered to optimize binding affinity, T-cell activation, and cytotoxicity while minimizing off-target effects (Usmani et al., 2021).

2. **Clinical Trials**

- **Phase 1 Trials (MagnetisMM-1)**: The first-in-human study of elranatamab (MagnetisMM-1) assessed safety, tolerability, and preliminary efficacy. Patients with relapsed or refractory multiple myeloma who had exhausted available treatments were enrolled. The trial showed encouraging response rates, with manageable adverse events such as cytokine release syndrome (CRS) and cytopenias (Usmani et al., 2021).

- **Phase 2 Trials (MagnetisMM-3)**: Building on the success of the phase 1 study, the MagnetisMM-3 trial expanded to evaluate elranatamab in a larger cohort of relapsed multiple myeloma patients. This trial confirmed the earlier findings, demonstrating substantial response rates and deep, durable remissions in some patients (Mateos et al., 2022).

- **Ongoing Trials (MagnetisMM-5)**: The ongoing MagnetisMM-5 trial is comparing elranatamab to standard of care therapies in relapsed multiple myeloma. Early results indicate continued strong efficacy, further establishing elranatamab as a promising treatment option (Usmani et al., 2023).

3. **Combination Therapies and Future Directions**

- **Combination Strategies**: Research is underway to evaluate elranatamab in combination with other anti-myeloma agents, including immunomodulatory drugs (IMiDs), proteasome inhibitors, and monoclonal antibodies, to enhance efficacy and overcome resistance mechanisms (Chari et al., 2023).

- **Future Research**: Long-term follow-up and real-world studies are essential to understand the durability of responses, long-term safety, and optimal sequencing of elranatamab within the treatment landscape of multiple myeloma.

#### Conclusion

Elranatamab has emerged as a promising therapeutic agent for relapsed multiple myeloma, leveraging the targeted approach of bispecific antibody technology to harness the immune system against myeloma cells. Early clinical trials have demonstrated its efficacy and safety, paving the way for its potential integration into standard treatment protocols. Ongoing research and combination strategies will further refine its use and improve outcomes for patients with relapsed multiple myeloma.

---

**References:**

1. Mateos, M. V., Krishnan, A., Avet-Loiseau, H., et al. (2022). MagnetisMM-3: A phase 2 trial of elranatamab (PF-06863135), a BCMA-targeted bispecific antibody, in patients with relapsed/refractory multiple myeloma. *Journal of Clinical Oncology*, 40(16_suppl), 8003.

2. Usmani, S. Z., Garfall, A. L., van de Donk, N. W. C. J., et al. (2021). MagnetisMM-1: Initial results from a phase 1 study of elranatamab (PF-06863135), a B-cell maturation antigen (BCMA)-CD3 bispecific antibody, in patients with relapsed/refractory multiple myeloma (RRMM). *Blood*, 138(Supplement 1), 161.

3. Usmani, S. Z., Mateos, M. V., Anderson, L. D., et al. (2023). MagnetisMM-5: A randomized phase 3 study of elranatamab versus standard of care in patients with relapsed/refractory multiple myeloma. *Blood*, 142(Supplement 1), 252.

4. Chari, A., Usmani, S. Z., Mateos, M. V., et al. (2023). Elranatamab: Potential combination strategies in relapsed/refractory multiple myeloma. *Journal of Clinical Oncology*, 41(15_suppl), TPS8062.

- 1. **Gemini Output**

**Elranatamab: A Novel Approach to Relapsed Multiple Myeloma**

Elranatamab, a BCMA-CD3 bispecific antibody, represents a significant advancement in the treatment of relapsed or refractory multiple myeloma (RRMM). Targeting BCMA, a protein overexpressed on myeloma cells, elranatamab directs T cells to eliminate malignant plasma cells.

The development of elranatamab was driven by the need for effective therapies for patients with RRMM who have exhausted available treatment options. Early studies demonstrated the potential of BCMA-targeted therapies in this setting, prompting the development of elranatamab.

The pivotal MagnetisMM-3 trial evaluated the efficacy and safety of elranatamab in patients with RRMM. Results from this trial demonstrated impressive response rates, including a high proportion of deep remissions, in patients who had previously received multiple lines of therapy, including other BCMA-directed therapies [1].

These findings position elranatamab as a promising treatment option for patients with RRMM, particularly those with limited therapeutic options. Ongoing research is focused on optimizing elranatamab's use, including exploring combination therapies and investigating its role in earlier lines of treatment.

In summary, elranatamab represents a novel and effective approach to the management of RRMM. Its development has significantly impacted the treatment landscape for patients with this challenging disease.

**Citation:**

1. San Miguel J, et al. Elranatamab in relapsed or refractory multiple myeloma: phase 2 MagnetisMM-3 trial results. Nat Med. 2023;29(10):1964-1972.
   1. **Llama 2 Output**

**Synopsis: Development and Evolution of Therapy with Elranatamab for Relapsed Multiple Myeloma**

Elranatamab, a bispecific antibody targeting BCMA and CD3, has emerged as a promising therapy for relapsed multiple myeloma (RMM).

**Early Developments**

- BCMA-targeting therapies showed efficacy in RMM [1].
- Bispecific antibodies, such as elranatamab, offered a novel approach to engage T-cells and target myeloma cells [2].

**Introduction of Elranatamab**

- The phase 1/2 MagnetisMM-1 trial investigated elranatamab in RMM, demonstrating high overall response rates (ORR) and complete response (CR) rates [3].
- The phase 2 MagnetisMM-2 trial confirmed elranatamab's efficacy in RMM, with improved ORR and CR rates [4].

**Evolution and Optimization**

- Ongoing trials are investigating elranatamab in combination with other novel agents, such as lenalidomide and pomalidomide, to enhance outcomes [5].
- Research is also exploring optimal dosing and scheduling, as well as biomarkers for treatment response.

**Current Status and Future Directions**

- Elranatamab has shown significant promise in RMM, offering a new treatment option for patients [6].
- Future studies will focus on optimizing elranatamab-based regimens, exploring its use in earlier lines of therapy, and investigating its potential in combination with other emerging therapies.

References:

[1] Lonial et al. (2018). BCMA-targeting therapies in multiple myeloma. *Blood*, 132(11), 1131-1141.

[2] Labrijn et al. (2019). Bispecific antibodies for cancer treatment. *Nature Reviews Drug Discovery*, 18(10), 767-786.

[3] Bahlis et al. (2022). Elranatamab in relapsed multiple myeloma: MagnetisMM-1. *Blood*, 139(11), 1551-1564.

[4] Usmani et al. (2022). Elranatamab in relapsed multiple myeloma: MagnetisMM-2. *Journal of Clinical Oncology*, 40(10), 1081-1091.

[5] (link unavailable) (2022). Elranatamab in combination with lenalidomide and pomalidomide in relapsed multiple myeloma. *NCT04954444*.

[6] Kumar et al. (2022). Multiple myeloma: diagnosis and treatment. *Mayo Clinic Proceedings*, 97(5), 851-874.

## Talquetamab

1. **Claude 3.5 Output**

Here's a synopsis of the development and evolution of therapy with talquetamab for relapsed multiple myeloma:

Talquetamab is a first-in-class bispecific antibody targeting both GPRC5D (G protein-coupled receptor family C group 5 member D) and CD3. It was developed as a novel immunotherapy approach for relapsed/refractory multiple myeloma (RRMM), particularly for patients who have exhausted other treatment options.

The rationale for targeting GPRC5D stems from its high expression on multiple myeloma cells and limited expression on normal tissues, potentially offering a therapeutic window with manageable toxicity [1].

Key milestones in the development of talquetamab include:

1. Preclinical studies: Initial research demonstrated the potent anti-myeloma activity of talquetamab in laboratory and animal models, providing the foundation for clinical development [2].

2. Phase 1 trial: The first-in-human study (MonumenTAL-1) established the safety and preliminary efficacy of talquetamab in heavily pretreated RRMM patients. This study explored both intravenous and subcutaneous administration routes and helped determine the recommended phase 2 dose [3].

3. Phase 2 trial: The MonumenTAL-1 study was expanded to include a phase 2 portion, further evaluating the efficacy and safety of talquetamab at the recommended dose [4].

The evolution of talquetamab therapy has focused on several key areas:

1. Dosing optimization: Studies have explored various dosing schedules, including weekly and biweekly administration, to balance efficacy and tolerability [5].

2. Subcutaneous administration: The development of a subcutaneous formulation has improved patient convenience and potentially reduced infusion-related reactions [6].

3. Combination strategies: Ongoing trials are investigating talquetamab in combination with other agents, including anti-CD38 antibodies and immunomodulators [7].

4. Patient selection: Research is ongoing to identify biomarkers that may predict response to talquetamab, including GPRC5D expression levels [8].

Key findings from clinical trials include:

1. Efficacy: High overall response rates (60-70%) have been reported in heavily pretreated RRMM patients, including those refractory to multiple prior lines of therapy [9].

2. Safety profile: The most common adverse events include cytokine release syndrome (CRS), skin-related toxicities, and nail disorders. These are generally manageable with supportive care and tend to occur early in treatment [10].

3. Duration of response: Preliminary data suggest durable responses, with median duration of response not reached in some cohorts at the time of reporting [11].

Ongoing research and future directions include:

1. Phase 3 trials: Larger, randomized studies comparing talquetamab to standard of care therapies are planned or underway [12].

2. Earlier lines of therapy: While initial development focused on heavily pretreated patients, there is growing interest in evaluating talquetamab in earlier lines of therapy [13].

3. Resistance mechanisms: Investigations into mechanisms of resistance to GPRC5D-targeted therapies and strategies to overcome them are ongoing [14].

4. Long-term follow-up: Continued monitoring of patients treated with talquetamab to assess long-term efficacy and safety [15].

5. Quality of life assessments: Given the unique toxicity profile of talquetamab, understanding its impact on patient-reported outcomes is crucial [16].

In conclusion, the development of talquetamab represents a significant advance in immunotherapy for RRMM, offering a novel target and promising efficacy in heavily pretreated patients. Its evolution continues as researchers refine its use, explore combination strategies, and investigate its potential across the multiple myeloma treatment landscape.

Would you like me to elaborate on any specific aspect of this synopsis or provide more information about talquetamab or bispecific antibodies in multiple myeloma?

1. **ChatGPT 4.0 Output**

### Synopsis for the Development and Evolution of Therapy with Talquetamab for Relapsed Multiple Myeloma

#### Introduction

Talquetamab is a novel bispecific antibody targeting GPRC5D and CD3, representing a promising new therapeutic approach for patients with relapsed or refractory multiple myeloma. By engaging T-cells and directing them to target GPRC5D-expressing myeloma cells, talquetamab has shown significant potential in early clinical trials. This synopsis outlines the development, clinical trials, and evolution of talquetamab therapy for relapsed multiple myeloma.

#### Development of Key Therapy

1. **Talquetamab (Talq)**

- **Mechanism of Action**: Talquetamab is a bispecific antibody that targets both GPRC5D, a receptor highly expressed on myeloma cells, and CD3 on T-cells. This dual targeting activates T-cells and redirects them to kill GPRC5D-expressing myeloma cells.

- **Clinical Trials**: Initial trials have demonstrated significant anti-myeloma activity with an acceptable safety profile in patients with relapsed or refractory multiple myeloma (Usmani et al., 2022).

#### Evolution of Therapy

1. **Preclinical Development**

- **Rationale and Design**: Preclinical studies identified GPRC5D as a promising target due to its restricted expression on malignant plasma cells and limited presence on normal tissues. Talquetamab was designed to exploit this target, offering a novel therapeutic strategy for multiple myeloma (Chari et al., 2021).

2. **Clinical Trials**

- **Phase 1 Trials (MonumenTAL-1)**: The first-in-human study, MonumenTAL-1, evaluated the safety, tolerability, and preliminary efficacy of talquetamab in relapsed or refractory multiple myeloma. The trial reported encouraging response rates with manageable adverse events, such as cytokine release syndrome (CRS) and cytopenias (Usmani et al., 2022).

- **Phase 2 Trials**: Building on the success of the phase 1 study, phase 2 trials further assessed the efficacy and safety of talquetamab in a larger cohort. These trials confirmed the earlier findings and demonstrated deep, durable responses in some patients (Mateos et al., 2022).

- **Ongoing Trials**: Ongoing studies are exploring talquetamab in combination with other anti-myeloma agents, aiming to enhance its efficacy and broaden its applicability in the treatment landscape of multiple myeloma (Kumar et al., 2023).

3. **Combination Therapies and Future Directions**

- **Combination Strategies**: Research is ongoing to evaluate talquetamab in combination with standard therapies, including proteasome inhibitors, immunomodulatory drugs (IMiDs), and monoclonal antibodies, to enhance response rates and overcome resistance mechanisms (Chari et al., 2023).

- **Future Research**: Long-term follow-up studies and real-world data are crucial to understanding the durability of responses, long-term safety, and optimal integration of talquetamab into existing treatment paradigms.

#### Conclusion

Talquetamab represents a significant advancement in the treatment of relapsed multiple myeloma, leveraging bispecific antibody technology to target GPRC5D-expressing myeloma cells. Early clinical trials have demonstrated its efficacy and manageable safety profile, paving the way for its potential incorporation into standard treatment regimens. Ongoing research and combination strategies will further refine its use and improve outcomes for patients with relapsed multiple myeloma.

---

**References:**

1. Usmani, S. Z., Garfall, A. L., van de Donk, N. W. C. J., et al. (2022). MonumenTAL-1: Initial results from a phase 1 study of talquetamab, a GPRC5D-CD3 bispecific antibody, in patients with relapsed/refractory multiple myeloma (RRMM). *Blood*, 138(Supplement 1), 161.

2. Mateos, M. V., Krishnan, A., Avet-Loiseau, H., et al. (2022). Phase 2 trial of talquetamab in relapsed/refractory multiple myeloma: Efficacy and safety outcomes. *Journal of Clinical Oncology*, 40(16_suppl), 8003.

3. Kumar, S., Mateos, M. V., Anderson, L. D., et al. (2023). Ongoing studies of talquetamab in combination therapies for relapsed/refractory multiple myeloma. *Blood*, 142(Supplement 1), 252.

4. Chari, A., Usmani, S. Z., Mateos, M. V., et al. (2023). Talquetamab: A novel bispecific antibody for the treatment of relapsed/refractory multiple myeloma. *Journal of Clinical Oncology*, 41(15_suppl), TPS8062.

1. **Gemini Output**

**Talquetamab: A Novel Approach to Relapsed Multiple Myeloma**

Talquetamab represents a significant advancement in the treatment of relapsed or refractory multiple myeloma (RRMM). This bispecific antibody targets both GPRC5D, a novel drug target on myeloma cells, and CD3 on T-cells, effectively redirecting the patient's immune system to attack cancer cells.

The development of talquetamab was driven by the urgent need for new therapies for patients with RRMM who have exhausted available treatment options. Early clinical studies demonstrated promising efficacy and safety, supporting its potential as a breakthrough treatment.

The pivotal MonumenTAL-1 study evaluated the safety and efficacy of talquetamab in patients with RRMM who had received multiple prior lines of therapy. Results from this study showed impressive response rates, including deep remissions, in heavily pre-treated patients [1]. These findings highlight talquetamab's potential to address the unmet medical needs of patients with RRMM.

As research continues, the role of talquetamab in the treatment landscape for RRMM is evolving. Further studies are investigating optimal dosing, combination therapies, and its potential use in earlier lines of treatment.

In conclusion, talquetamab represents a novel and effective approach to the management of RRMM. Its development has significantly impacted the treatment landscape for patients with this challenging disease.

**Citation:**

1. [Include relevant citation for MonumenTAL-1 study]
2. **Llama 2 Output**

**Synopsis: Development and Evolution of Therapy with Talquetamab for Relapsed Multiple Myeloma**

Talquetamab, a bispecific antibody targeting GPRC5D and CD3, has emerged as a promising therapy for relapsed multiple myeloma (RMM).

**Early Developments**

- GPRC5D was identified as a target in multiple myeloma [1].
- Bispecific antibodies, such as talquetamab, offered a novel approach to engage T-cells and target myeloma cells [2].

**Introduction of Talquetamab**

- The phase 1/2 MonumenTAL-1 trial investigated talquetamab in RMM, demonstrating high overall response rates (ORR) and complete response (CR) rates [3].
- The phase 2 MonumenTAL-2 trial confirmed talquetamab's efficacy in RMM, with improved ORR and CR rates [4].

**Evolution and Optimization**

- Ongoing trials are investigating talquetamab in combination with other novel agents, such as pomalidomide and dexamethasone, to enhance outcomes [5].
- Research is also exploring optimal dosing and scheduling, as well as biomarkers for treatment response.

**Current Status and Future Directions**

- Talquetamab has shown significant promise in RMM, offering a new treatment option for patients [6].
- Future studies will focus on optimizing talquetamab-based regimens, exploring its use in earlier lines of therapy, and investigating its potential in combination with other emerging therapies.

References:

[1] Lohr et al. (2019). GPRC5D as a target in multiple myeloma. *Blood*, 134(11), 933-943.

[2] Labrijn et al. (2019). Bispecific antibodies for cancer treatment. *Nature Reviews Drug Discovery*, 18(10), 767-786.

[3] Berdeja et al. (2022). Talquetamab in relapsed multiple myeloma: MonumenTAL-1. *Blood*, 139(11), 1565-1576.

[4] Chari et al. (2022). Talquetamab in relapsed multiple myeloma: MonumenTAL-2. *Journal of Clinical Oncology*, 40(10), 1092-1102.

[5] (link unavailable) (2022). Talquetamab in combination with pomalidomide and dexamethasone in relapsed multiple myeloma. *NCT05068920*.

[6] Kumar et al. (2022). Multiple myeloma: diagnosis and treatment. *Mayo Clinic Proceedings*, 97(5), 851-874.

**Survey Tool for LLM Output Evaluation**

| **Accuracy:** How accurate is the synopsis generated by the LLM? | 1. Completely inaccurate  2. Inaccurate  3. Neutral  4. Accurate  5. Perfectly accurate |
| --- | --- |
| **Completeness:** Assess whether all relevant information is included. | 1. Completely incomplete  2. Incomplete  3. Neutral  4. Relevant  5. Perfectly Relevant |
| **Relevance :** Assess whether the information provided is pertinent to the regimen. | 1. Completely irrelevant  2. Irrelevant  3. Neutral  4. Relevant  5.Perfectly relevant |
| **Clarity:** Assess how clearly the information is presented. | 1. Completely unclear  2. Unclear  3. Neutral  4. Clear  5. Very clear |
| **Hallucinations:** Assess to what degree hallucinations judged to be present | 1. Many hallucinations  2. Some hallucinations  3. Few hallucinations  4. No hallucinations |
| **Coherence:** Assess how well the output maintains topic and argument structure across a text. | 1. Completely incoherent  2. Incoherent  3. Neutral  4. Coherent  5. Completely coherent |
| I feel that the synopsis generated by the LLM would only require minimal extra editing before it could be more widely used. | 1. True  2. False |
| Please provide narrative comments about the synopsis,  if applicable. |  |

#

# Narrative Comments

Physician Reviewer 1:

|  | Claude | GPT-4 | Gemini | Llama |
| --- | --- | --- | --- | --- |
| Dara-RVD | Very close to the mark, would benefit from more information about safety. | Inclusion of GEM-CESAR, which is not an NDMM paper and not a paper investigating Dara-RVd, is a major red flag in this synopsis. CASTOR is also not an NDMM paper and not relevant. PERSEUS is also not included. | PERSEUS should be included. | Many inaccuracies - GRIFFIN does not investigate Dara-Rd, CASSIOPEIA is not a trial comparing Dara-Rd and RVd, and the conclusions are not oriented around Dara-RVd. |
| KRD | Generally pretty good. One minor inaccuracy - CHAMPION does not investigate KRd. But requires minimal adjustment. | FORTE is not mentioned, ASPIRE is alluded to twice, also is somewhat vague with respect to what is meant by "efficacy". | The primary pivotal trials of KRd in management of NDMM (Jakubowiak, ENDURANCE, FORTE, etc) | Not clear KRd was more tolerable than RVd on ENDURANCE, just different AEs. Also FORTE is not a trial comparing different dosing schedules of carfilzomib. |
| VTD-PACE | Excellent synopsis | Generally accurate, less comprehensive than the first LLM, somewhat innacurate with respect to the likely MoA of thalidomide. | Insufficient detail, incomplete, however generally accurate (although not perfectly so because of the statement made about VTD-PACE not being investigated frontline, which it is) |  |
| Dara-VCD | Some minor issues - dara was subQ all along in ANDROMEDA - but generally accurate. Should specify it's only studied in I-IIIA patients | As with prior iterations with the second LLM - too much mechanistic focus | Brief and generally accurate with few hallucinations, but needs more expansion on the ANDROMEDA results. |  |
| Elranatamab | Little about safety included | A bit much mechanistically, also nothing on safety. | Also nothing about safety in this synopsis. Reference is not the correct reference and somewhat misleading (cohort B not presented in the main elranatamab manuscript) and efficacy in that setting is lower. | Really thin on detailed information, also misleading to say that elra led to "improved" ORR and CR rates in MagnetisMM-3, also MagnetisMM-2 is not a published study. |
| Talquetamab | Somewhat misleading on the safety profile, the expression of efficacy is somewhat duplicative with itself. | Not sure what that Mateos abstract is referring to - I can't find it - but it's definitely not a confirmatory study of MonumenTAL-1. The toxicity profile is also not entirely accurate. These remain relatively minor edits. | Superficial compared to other abstracts and does not really discuss safety. | Not aware the MonumenTAL-2 study exists or has been published, MonumenTAL-1 is the pivotal study and is not cited here, the Tal-Pd study alluded to does not have a correct NCT code and I cant find it, so it may not exist. |

Physician Reviewer 2:

|  | Claude | GPT-4 | Gemini | Llama |
| --- | --- | --- | --- | --- |
| Dara-RVD | Only critique is that PERSEUS is published and not just an ASH abstract, but the fact that it knew that ASH means presentation is impressive. | No mention of PERSEUS. GEM-CESAR of course doesn't belong here (it's a smoldering myeloma trial) and didn't use D-VRd, which I'd say is a critical hallucination. | No mention of the PERSEUS trial. This type of flowery language is a dead giveaway for AI-generated text: "This combination therapy offers a beacon of hope for those battling MM." | The worst of the 4. Wrong prompt (Dara-Rd being discussed, not Dara-VRd), incorrect information regarding CASSIOPEIA and GRIFFIN (including others), and incorrect citations. |
| KRD | Ideally a mention of ENDEAVOR might have been nice to explain the K > V argument in RRMM, but otherwise well written. | ENDURANCE discussion could be a bit more thorough. I think the Korde KRd citation is incorrect, or perhaps an ASCO abstract and not a publication? | Both completely made up, and classic AI chatbot language like "KRd offers a powerful option for those battling this challenging condition." | I'd say this statement is inaccurate: "demonstrating similar efficacy but improved tolerability with KRd" with ENDURANCE. The FORTE trial wasn't designed to focus on different K dosing schedules (although they did change KR maintenance dosing later), and the citation for Dr. Gay's paper should be Lancet Oncol. |
| VTD-PACE | It would be nice to mention KPD-PACE, but the fact that they suggested VRD-PACE as a newer point is encouraging. | It would be nice to mention KPD-PACE, but the fact that they suggested VRD-PACE as a newer point is encouraging. | Interestingly, because there aren't as many studies of this regimen, this time this one did a good job. I'm glad they mentioned the side effects of VTD-PACE. This line remains a classic AI-style line: "offering hope for patients battling advanced MM." | Citations and premise of how VTD-PACE were developed is entirely made up. |
| Dara-VCD | Very little to add. | Even the ANDROMEDA citation is incorrect, but otherwise the text is okay. | This sentence reads oddly: "Additionally, the long-term effects of DaraVCD on organ damage and overall survival are being closely monitored." I don't think D-CyBorD is causing organ damage per se. The citation for ANDROMEDA is wrong again. | Too generic, and what is written is largely inaccurate. |
| Elranatamab | Little about safety included | A bit much mechanistically, also nothing on safety. | Also nothing about safety in this synopsis. Reference is not the correct reference and somewhat misleading (cohort B not presented in the main elranatamab manuscript) and efficacy in that setting is lower. | Really thin on detailed information, also misleading to say that elra led to "improved" ORR and CR rates in MagnetisMM-3, also MagnetisMM-2 is not a published study. |
| Talquetamab | The toxicity profile is underplayed, e.g. tongue / taste toxicities not brought up. The text at the bottom is a dead giveaway that this is AI. | No mention of toxicities. Citations are inaccurate and also don't include the actual publications. | Too vague, no mention of toxicities, no citations listed. This "1. [Include relevant citation for MonumenTAL-1 study]" would be a dead giveaway that it's AI (or a sloppy human in real life). | Vague, no mention of toxicities, citations are incorrect. |
